# Association between psychological distress trajectories from adolescence to midlife and mental health during the pandemic: evidence from two British birth cohorts

**DOI:** 10.1017/S0033291722003877

**Published:** 2023-10

**Authors:** V. Moulton, A. Sullivan, P. Patalay, E. Fitzsimons, M. Henderson, D. Bann, G. B. Ploubidis

**Affiliations:** 1 Centre for Longitudinal Studies, University College London, London, UK; 2 MRC Unit for Lifelong Health, University College London, London, UK

**Keywords:** COVID-19, life satisfaction, life-course, loneliness, mental health, midlife, psychological distress, trajectories

## Abstract

**Background:**

This paper examined whether distinct life-course trajectories of psychological distress from adolescence to midlife were associated with poorer mental health outcomes during the pandemic.

**Methods:**

We present a secondary analysis of two nationally representative British birth cohorts, the 1958 National Child Development Study (NCDS) and 1970 British Cohort Study (BCS70). We used latent variable mixture models to identify pre-pandemic longitudinal trajectories of psychological distress and a modified Poisson model with robust standard errors to estimate associations with psychological distress, life satisfaction and loneliness at different points during the pandemic.

**Results:**

Our analysis identified five distinct pre-pandemic trajectories of psychological distress in both cohorts. All trajectories with prior symptoms of psychological distress irrespective of age of onset, severity and chronicity were associated with a greater relative risk of poorer mental health outcomes during the pandemic and the probability of poorer mental health associated with psychological distress trajectories remained fairly constant. The relationship was not fully attenuated when most recent pre-pandemic psychological distress and other midlife factors were controlled for.

**Conclusions:**

Whilst life-course trajectories with any prior symptoms of psychological distress put individuals at greater risk of poor mental health outcomes during the pandemic, those with chronic and more recent occurrences were at highest risk. In addition, prior poor mental health during the adult life-course may mean individuals are less resilient to shocks, such as pandemics. Our findings show the importance of considering heterogeneous mental health trajectories across the life-course in the general population in addition to population average trends.

## Introduction

Globally, evidence shows as a result of the pandemic poorer mental health has increased (Santomauro et al., 2021). However there have been mixed findings, in the early stages of the pandemic, some studies showed significantly increased levels of poor mental health in the general population (Daly, Sutin, & Robinson, 2022; Fancourt, Steptoe, & Bu, 2021; Pierce et al., 2020). A recent meta-analysis of longitudinal studies shows overall (but with a high degree of heterogeneity across samples) mental health symptoms became comparable to pre-pandemic levels by mid-2020 in Europe and North America (Robinson, Sutin, Daly, & Jones, 2022), while Patel et al. (2022) using 11 longitudinal cohorts in the UK indicate on average mental health in the general population has deteriorated since the onset of the pandemic with no evidence of recovery.

It is important to identify and examine populations who may be at greater risk of poorer mental health, including those with pre-existing and a history of poorer mental health. There have been fewer studies investigating individuals with pre-existing mental health conditions. As with the general population, the evidence is also mixed, some studies suggest worsening mental health in the early stages of the pandemic (Fancourt et al., 2021; O'Connor et al., 2021; Pierce et al., 2020), while other studies show no increase in symptoms (Daly et al., 2022; Robinson et al., 2022) and a graded dose–response relation based on prior number of symptoms and chronicity (Pan et al., 2021). Also differences have been found in the impact of the pandemic across diagnoses, with anxiety, depression, post-traumatic stress disorder and eating disorders at high risk of worsened mental health (Lewis et al., 2022).

Prior studies have tended to employ retrospective measures of pre-existing mental health. Even in prospective studies, pre-COVID-19 mental health data have typically been measured at one time-point only or over the short-term (Daly et al., 2022; Pan et al., 2021; Pierce et al., 2020; Robinson et al., 2022). However, no extant studies have investigated life-course trajectories of mental health in the general population prior to the COVID-19 outbreak (Kessler et al., 2005; Moffitt et al., 2010).

The risk of poor mental health in the general population has been shown to be heterogeneous, following different longitudinal trajectories in terms of number, ranging from 3 to 6 classes, characterised by age of onset, symptom severity and risks of recurrence (Colman, Ploubidis, Wadsworth, Jones, & Croudace, 2007; Musliner, Munk-Olsen, Eaton, & Zandi, 2016; Paksarian et al., 2016). Most studies identify one or more stable low or no symptoms group, a class with persistently high symptoms, and groups of unstable symptoms either decreasing or increasing over time and others also finding a moderate stable symptoms group (Musliner et al., 2016). Distinct pre-pandemic life-course mental health trajectories based on onset, severity and stability across the adult life-span may also be related to different levels of poor mental health during the pandemic.

Mental well-being is distinct from mental illness (Keyes, 2005) and predicts important outcomes including physical health. Like mental health, there were mixed findings on wellbeing during COVID-19 (Prati & Mancini, 2021; Zacher & Rudolph, 2021). Life satisfaction is sometimes favoured by governments as positive measures are deemed better for evaluating public health approaches (Frijters, Clark, Krekel, & Layard, 2020). Furthermore, the pandemic and measures used to mitigate the crisis may have a disproportionate influence on those with mental health difficulties because of increased isolation and loss of social connectedness (Santini et al., 2020). Indeed, a recent systematic review of loneliness during the COVID-19 pandemic shows increased levels of loneliness, especially in relation to age (older and younger) and clinical populations (Ernst et al., 2022). Loneliness is a key predictor of mental health challenges particularly in older adults (Luchetti et al., 2020).

This study extends the literature by examining the heterogeneity in pre-pandemic mental health across the adult life-course in the general population, and not just focusing on average differences across two time-points or a single diagnosis/poorer mental health at one point pre-pandemic. This is important as it may be that individuals with poorer mental health at different stages of the life-course may be less resilient to sudden shocks. In addition, unlike most other studies we investigate mental health during a number of phases of the pandemic, not just in Spring and Summer 2020. Also, we take account of early-life confounders and midlife mediators by exploiting the rich longitudinal data available in two large nationally representative British birth cohorts from birth and across the life-course to investigate whether, (i) differing pre-pandemic psychological distress trajectories in these populations were more likely to result in symptoms of psychological distress, lower life satisfaction and feelings of loneliness during the COVID-19 pandemic, (ii) whether there were differences in these mental health outcomes in later midlife, for life-course psychological distress trajectories at three distinct time-points, from May 2020 through to March 2021, as the pandemic developed and (iii) whether poor mental health over the life-course is associated with being less resilient to pandemic-related shocks.

## Method

### Participants

Our data are from two ongoing population-based birth cohorts:

- *1958 National Child Development Study* (*NCDS):* The NCDS follows the lives of 17 415 people that were born in England, Scotland or Wales in a single week in March 1958. The NCDS started as the Perinatal Mortality Survey and captured 98% of the total births in Great Britain in the target week. The cohort has been followed up eleven times between ages 7 and 55 (Power & Elliott, 2006).
- *1970 British Cohort Study* (*BCS70):* The BCS70 follows the lives of 17 198 people (representing 95–98% of the target population) born in England, Scotland and Wales in a single week in April 1970. Participants have since been followed up nine times between ages 5 and 46 (Elliott & Shepherd, 2006; Sullivan, Brown, Hamer, & Ploubidis, 2022).

In addition, during the COVID-19 pandemic participants of the NCDS and BCS70 completed an online survey at three different time-points when they were aged 62 and 50, respectively. The first survey was conducted during the first national lockdown, between 4 and 26 May 2020 (Wave 1: NCDS *N*: 5178; BCS70 *N*: 4223). The second survey was completed between 10 September and 16 October 2020 (Wave 2: NCDS *N*: 6282; BCS70 *N*: 5320) when the first national lockdown had been lifted, but restrictions on social contact still remained, and the third survey was conducted during the third national lockdown, between 1 February and 21 March 2021 (Wave 3: NCDS *N*: 6757; BCS70 *N*: 5684) (Brown et al., 2021). Ethical approval for the NCDS and BCS70 has been granted by the National Health Service (NHS) Research Ethics Committee and all participants have given informed consent.

Our analytic sample includes all participants in the NCDS and BCS70 surveys, excluding those who had died or emigrated by age 50 in the NCDS and age 46 in the BCS70. The sample size for NCDS is *n* = 15 291 and for BCS70 *n* = 16 128 (sample descriptive statistics are presented in online Supplementary Table S1 and cohort member response at each relevant sweep in online Supplementary Table S2). To deal with attrition and item non-response and to restore sample representativeness we used Multiple Imputation (MI) with chained equations, imputing 25 data sets separately for both cohorts at each wave of the COVID-19 surveys (Little & Rubin, 2019). All variables used in our main analysis, as well as a set of auxiliary variables (including variables related to non-response to the COVID-19 surveys) were included in the imputation models to maximise the plausibility of the ‘missing at random’ (MAR) assumption in order to reduce bias due to missing data (Mostafa et al., 2021; Silverwood, Narayanan, Dodgeon, & Ploubidis, 2020). Due to non-response and to increase power we included imputed outcomes in our analysis (Kontopantelis, White, Sperrin, & Buchan, 2017; Madley-Dowd, Hughes, Tilling, & Heron, 2019), and as an additional sensitivity analysis all models were rerun in line with the ‘impute and delete’ method (Von Hippel, 2007) and resulted in similar findings (available in online Supplementary Table S3).

### Measures

#### Pre-pandemic psychological distress

Psychological distress was measured in both cohorts with the nine-item version of the Malaise Inventory (Rodgers, Pickles, Power, Collishaw, & Maughan, 1999; Rutter, Tizard, & Whitmore, 1970) from ages 23, 33, 42 and 50 in the NCDS and ages 26, 34, 42 and 46 in the BCS70. Psychological distress captures depression and anxiety symptoms (Drapeau, Marchand, & Beaulieu-Prévost, 2012). In both surveys the Malaise items were assessed via written self-completion, either on paper or via computer. The Malaise inventory has been shown to have good psychometric properties (McGee, Williams, & Silva, 1986), measurement invariance (Ploubidis, McElroy, & Moreira, 2019), and has been used in general population studies as well as investigations of high-risk groups (Furnham & Cheng, 2015). In both cohorts, at age 16 four items from the Children's Behavior Questionnaire (CBQ) (Rutter et al., 1970) reflective of affective disorders (low mood, irritability, worry and fearfulness), as reported by the child's mother were employed.

#### Outcomes

There were three outcome measures, psychological distress, life satisfaction and loneliness, completed at three time-points during the pandemic. Psychological distress was measured in both cohorts with the nine-item version of the Malaise Inventory (Rodgers et al., 1999), the same measure assessed across the life-course pre-pandemic (Wave 1: Cronbach's *α* = 0.77; Wave 2: *α* = 0.79; Wave 3: *α* = 0.79). The measure was transformed into a dichotomous variable where 1 represents high psychological distress (rating ≥ 4). Subjective life satisfaction was measured by asking ‘Overall, how satisfied are you with your life nowadays, where 0 means ‘not at all’ and 10 means ‘completely’?’ (Office for National Statistics (ONS), 2020). Research has shown that single item measures of life satisfaction are highly correlated with longer life satisfaction scales (Cheung & Lucas, 2014). The measure was transformed into a dichotomous variable where 1 is high or very high level of satisfaction [rating ≥ 7 (ONS, 2018)]. Loneliness was measured using the three-item short form of the Revised UCLA loneliness scale (Hughes, Waite, Hawkley, & Cacioppo, 2004). Items were assessed on a three-point scale to produce a loneliness score from 3 to 9 (Wave 1: *α* = 0.88; Wave 2: *α* = 0.83; Wave 3: *α* = 0.82), a dichotomous variable was created using a cut-off score of 6 to represent presence of loneliness (Steptoe, Shankar, Demakakos, & Wardle, 2013). As sensitivity analysis we estimated models with continuous versions of all outcomes with appropriate link functions and transformations where possible leading to similar results (available in online Supplementary Table S7).

#### Potential confounders

We include in our analysis a rich set of variables comprising early life factors (sex, ever breastfed, mother smoked daily during pregnancy, gestation period and birthweight), socio-economic factors (parental social class, education, housing tenure, access to amenities, crowding and marital status), parental factors (maternal age at birth, mother worked at all in first 5 years, separated from child and read to), child behaviour and health [cohort member bedwetting since age 5, had any medical conditions and body mass index (BMI)], and cognitive ability (details are available in online Supplementary Table S4).

#### Mediators during midlife

In our regression-based mediation analysis we include a wide range of midlife (at age 50/55 in the NCDS and age 46 in BCS70) explanatory variables covering, adult social class, educated to degree level or not, income, employment status, housing tenure, household size, number of children, partnership status, midlife cognitive function, general health, ever smoked, problem drinking, BMI and the most recent pre-pandemic measure of psychological distress at age 50 in the NCDS and age 46 in the BCS70 (details are available in online Supplementary Table S4).

### Analytic approach

As a first step, we used latent variable mixture models to identify longitudinal typologies of mental health (Colman et al., 2007) employing psychological distress at five age equivalent time-points in both cohorts from adolescence to midlife (Rodgers et al., 1999). For each of the latent measures of psychological distress we conducted confirmatory factor analysis, and derived a four-category ordinal variable on the basis of factor scores distributions of the latent measures (the first from the 1st to 50th percentile on the factor scores, the second from the 51st to the 75th, the third group individuals from the 76th to 90th and the fourth from the 91st to 100th percentile) to each of the five time-points in each cohort. Thus, each latent measure was recoded to a four-category ordinal variable at each of the five age equivalent time-points. We use latent class analysis to predict class membership, based on individual's posterior probability of belonging to each class, after random starting draws. Identification of latent classes was based on theory and research including prior work on trajectories of mental health (Colman et al., 2007; Musliner et al., 2016; Paksarian et al., 2016), including work using national cohorts (Gondek et al., 2022) and timing of onset in the general population (Kessler et al., 2007), as well as identifying the best-fitting model based on information criteria, accuracy, substantive meaning and parsimony (Bauer & Curran, 2003). (Further details are available in online Supplementary Tables S5.1 and S5.2.)

We first examined the proportion of individuals in each of the mental health classes prior to the pandemic with psychological distress, life-satisfaction and loneliness during the pandemic. We used a modified Poisson model with robust standard errors that returns risk ratios for ease of interpretation and to avoid bias due to non-collapsibility of the odds ratio (Pang, Kaufman, & Platt, 2016) to estimate associations between the psychological distress trajectories and mental health outcomes during the pandemic.

To aid interpretation we present between trajectories comparisons of marginal effects on psychological distress, life satisfaction and loneliness at three time-points during the pandemic, thereby answering question 2. To answer question 3, we re-ran the modified Poisson models and controlled for a variety of socio-economic, household, cognitive and health factors in midlife and then included the most recent pre-pandemic measure of psychological distress, thus investigating whether pre-pandemic psychological distress trajectories were associated with post-pandemic mental health over and above other factors with any remaining difference between trajectories thus attributed to the impact of the pandemic. All Latent mixture modelling was conducted using MPLUS v8.2, and descriptive analyses, GLM Poisson regression and multiple imputation were performed using Stata version 15 (StataCorp).

## Results

### Trajectories of psychological distress from adolescence to midlife

**Fig. 1.**
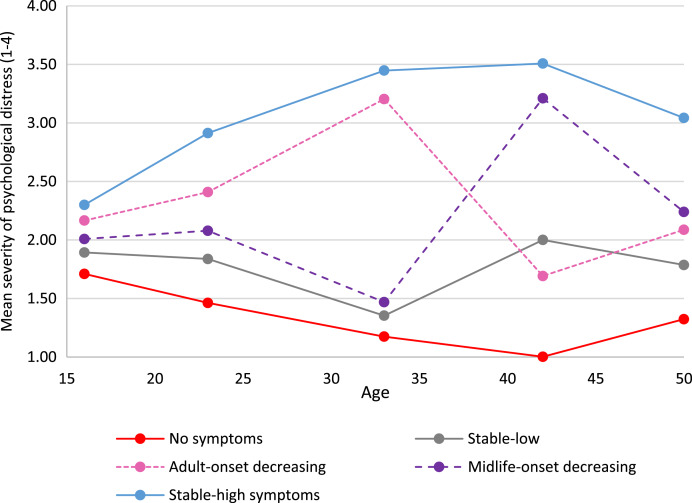
Five longitudinal classes of psychological distress from age 16 to 50 in the NCDS.

**Fig. 2.**
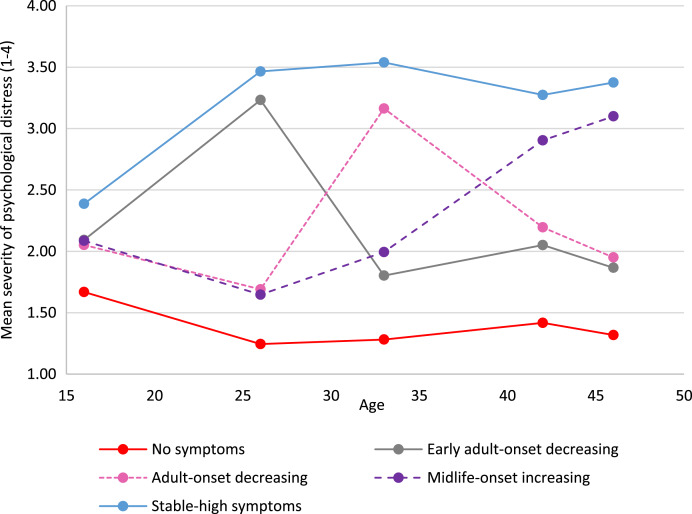
Five longitudinal classes of psychological distress from age 16 to 46 in the BCS70.

In both cohorts we identified five longitudinal trajectories (latent classes) of psychological distress as the most parsimonious models (online Supplementary Tables S5.1 and S5.2). Figures 1 and 2 show the means for each longitudinal latent class on each of the five measures of psychological distress in the NCDS (age 16–50) and BCS70 (age 16–46).

Based on observation the five longitudinal trajectories looked similar, but not identical in the two cohorts. The trajectory referents were defined on the basis of life-stage onset (i.e. age 20–30 ‘early adulthood’, age 31–40 ‘adulthood’, age 40+ ‘midlife’), severity (i.e. mean of <2 ‘low’, 2–3 ‘moderate’ and >3 ‘high’) and longevity (stable across 3 or more time-points). On all tables throughout this paper the order of the trajectory groups is firstly ‘no-symptoms’ and lastly ‘stable-high symptoms’, with other groups ordered by earliest life-stage increase in psychological distress.

The largest trajectory had few or no symptoms [‘no symptoms’, NCDS *n* = 6255 (40.9%); BCS70 *n* = 8515 (52.8%)]. There was also a trajectory with persistent severe symptoms [‘stable-high symptoms’, NCDS *n* = 3047 (19.9%); BCS70 *n* = 3113 (19.3%)], and a trajectory with adult-onset and more favourable outcomes [‘adult-onset decreasing’, NCDS n= 1658 (10.8%); BCS70 n= 1774 (11.0%)]. Both cohorts also had a trajectory with severe symptoms developing in midlife; however, in the NCDS the outcome was more positive [‘midlife-onset decreasing *n* = 1818 (11.9%)] whereas in the BCS70 the symptoms were increasing [‘midlife-onset increasing’, *n* = 1258 (7.8%)]. The final trajectory in the NCDS was repeated minor symptoms [‘stable-low symptoms’, *n* = 2513 (16.4%)], while in the BCS70 early adult onset with more favourable outcomes [‘early adult-onset decreasing’ BCS70 n= 1468 (9.1%)].

### Descriptive statistics of mental health outcomes during the pandemic

Descriptive statistics of the trajectories of psychological distress and mental health outcomes during the pandemic are presented in Table 1. During the first national lockdown in May 2020, overall, 17.5% and 22.7% (mean scores available in online Supplementary Table S6) of cohort members in the NCDS and BCS70 respectively were associated with high psychological distress, and 27.1% and 27.3% with loneliness, while the majority (67.4% in the NCDS and 64.4% in the BCS70) had high levels of life satisfaction. This pattern was broadly similar throughout the course of the pandemic (when lockdown restrictions were lifted and during the third lockdown).

**Table 1.** Percentage of cohort members with high psychological distress, high life satisfaction and feelings of loneliness by pre-pandemic psychological distress trajectories in the NCDS and BCS70 during the COVID-19 pandemic

	Psychological distress (% ≥4)			Life satisfaction (% ≥7)			Loneliness (% ≥6)		
	Wave 1 % (s.e.) [95% CIs]	Wave 2 % (s.e.) [95% CIs]	Wave 3 % (s.e.) [95% CIs]	Wave 1 % (s.e.) [95% CIs]	Wave 2 % (s.e.) [95% CIs]	Wave 3 % (s.e.) [95% CIs]	Wave 1 % (s.e.) [95% CIs]	Wave 2 % (s.e.) [95% CIs]	Wave 3 % (s.e.) [95% CIs]
**NCDS mental health trajectories**									
**Total (*n* = 15 291)**	17.7 (0.02) [14.7–20.8]	20.4 (0.01) [18.9–22.1]	21.7 (0.01) [20.0–23.4]	67.4 (0.02) [63.7–71.3]	68.6 (0.01) [66.5–70.7]	62.6 (0.01) [60.6–64.6]	27.1 (0.02) [24.0–30.2]	24.1 (0.01) [22.4–25.9]	30.7 (0.01) [28.6–32.8]
**No symptoms (*n* = 6255)**	3.7 (0.01) [2.6–4.7]	5.6 (0.01) [4.3–6.9]	5.6 (0.01) [4.5–6.7]	77.9 (0.02) [74.4–81.4]	79.7 (0.01) [77.6–81.8]	73.3 (0.01) [71.3–75.3]	14.5 (0.01) [11.8–17.2]	12.9 (0.01) [10.9–14.9]	17.0 (0.01) [15.1–18.8]
**Stable-low symptoms (*n* = 2513)**	9.4 (0.01) [6.8–12.1]	10.8 (0.01) [9.1–12.4]	12.7 (0.01) [10.7–14.6]	73.0 (0.02) [69.7–76.2]	75.2 (0.01) [72.6–77.9]	64.9 (0.02) [61.6–68.1]	21.9 (0.01) [18.8–24.9]	19.9 (0.01) [17.4–22.3]	27.1 (0.02) [23.8–30.4]
**Adult-onset decreasing (*n* = 1658)**	18.0 (0.02) [13.0–23.0]	21.0 (0.02) [16.6–25.4]	23.1 (0.02) [19.3–27.0]	65.5 (0.03) [59.4–71.7]	63.3 (0.02) [59.0–67.6]	59.0 (0.02) [54.9–63.2]	26.0 (0.02) [20.9–31.0]	25.0 (0.02) [20.4–29.4]	35.1 (0.02) [31.4–39.1]
**Midlife-onset decreasing (*n* = 1818)**	23.3 (0.03) [17.9–28.8]	24.3 (0.02) [21.0–27.6]	25.3 (0.02) [21.4–29.2]	63.0 (0.02) [57.9–68.0]	61.7 (0.02) [57.0–65.4]	58.7 (0.02) [55.2–62.1]	36.4 (0.03) [31.1–41.8]	32.1 (0.02) [28.5–35.6]	38.1 (0.02) [34.4–41.8]
**Stable-high symptoms (*n* = 3047)**	50.0 (0.04) [41.0–59.0]	56.9 (0.02) [52.6–61.2]	59.3 (0.02) [54.9–63.6]	45.3 (0.03) [38.2–52.4]	47.4 (0.03) [42.1–52.8]	43.1 (0.02) [38.8–47.4]	52.2 (0.03) [45.5–59.0]	45.8 (0.02) [41.3–50.3]	55.4 (0.02) [50.9–59.8]
**BCS70 mental health trajectories**									
**Total (*n* = 16 128)**	22.7 (0.01) [20.4–25.0]	26.1 (0.01) [24.6–27.7]	27.0 (0.01) [24.7–29.2]	64.4 (0.02) [60.8–68.2]	61.6 (0.01) [59.6–63.6]	59.0 (0.02) [55.7–62.3]	27.3 (0.01) [24.4–30.1]	30.9 (0.01) [28.7–33.1]	31.1 (0.01) [28.7–33.6]
**No symptoms (*n* = 8515)**	5.4 (0.01) [4.1–6.8]	7.1 (0.01) [5.8–8.3]	8.5 (0.01) [7.1–10.0]	76.6 (0.02) [73.1–80.0]	75.6 (0.01) [73.7–77.6]	70.4 (0.01) [67.4–73.2]	15.4 (0.01) [13.0–17.7]	17.3 (0.01) [15.0–19.5]	18.7 (0.01) [16.4–20.9]
**Early adult-onset decreasing (*n* = 1468)**	18.8 (0.02) [14.4–23.1]	22.6 (0.02) [18.2–27.0]	23.9 (0.02) [24.9–27.8]	64.9 (0.03) [58.1–71.7]	63.4 (0.02) [59.9–66.9]	58.7 (0.02) [53.7–63.7]	31.4 (0.03) [26.2–36.7]	30.7 (0.02) [25.7–35.7]	33.7 (0.02) [29.3–38.2]
**Adult-onset decreasing (*n* = 1774)**	22.0 (0.03) [16.7–27.2]	27.0 (0.02) [22.4–31.4]	27.7 (0.03) [22.3–33.1]	62.6 (0.03) [55.6–69.4]	57.6 (0.03) [52.4–62.8]	55.6 (0.03) [50.0–61.2]	31.5 (0.03) [26.2–36.8]	34.1 (0.03) [29.0–39.1]	35.5 (0.03) [29.6–41.5]
**Midlife-onset increasing (*n* = 1258)**	43.4 (0.03) [37.7–49.2]	48.4 (0.02) [43.9–52.8]	49.3 (0.02) [45.5–53.2]	51.5 (0.03) [46.2–56.8]	47.7 (0.02) [43.0–52.4]	48.0 (0.02) [43.2–52.7]	38.0 (0.03) [32.6–43.4]	41.9 (0.02) [37.4–46.4]	40.4 (0.02) [36.0–44.7]
**Stable-high symptoms (*n* = 3113)**	63.3 (0.03) [56.6–70.2]	70.4 (0.02) [66.6–74.4]	70.1 (0.02) [65.2–75.1]	37.4 (0.03) [30.8–44.1]	30.4 (0.02) [26.1–34.8]	34.2 (0.03) [27.7–40.7]	50.9 (0.03) [44.2–57.7]	61.9 (0.02) [57.6–66.2]	58.4 (0.02) [53.8–63.1]

Reported as Percentage (%); standard error (s.e.); confidence intervals [95% CIs].

Wave 1 (May 2020), Wave 2 (September-October 2020) and Wave 3 (February–March 2021).

Nevertheless, different levels of mental health during the pandemic were related to pre-pandemic trajectories of psychological distress (by age of onset and symptom severity). In total, 3.7% and 5.4% of the ‘no symptoms’ trajectory in the NCDS and BCS70 respectively, experienced high psychological distress early during the pandemic, compared to 9.4–50% in the NCDS and 18.8–63.3% in the BCS70 for other prior life-course mental health trajectories that had experienced pre-pandemic psychological distress symptoms at least once. Likewise, in both cohorts the proportion of individuals with feelings of loneliness and lower life satisfaction during the pandemic varied by trajectories of psychological distress; although the highest proportions were associated with ‘stable-high symptoms’, those with onset at different stages in adulthood and with varying levels of psychological distress prior to the pandemic were related to higher proportions of poorer mental health outcomes during the pandemic, than those with no prior symptoms.

### Risk of poor mental health during the pandemic by trajectories of psychological distress

Table 2 presents the relative risks in the fully adjusted models, associated with each of the mental health outcomes during the pandemic for different trajectories of psychological distress, with the largest trajectory ‘no symptoms’ used as the reference category in the analysis (additional analyses comparing alternative reference groups are available in online Supplementary Tables S11.1 and S11.2).

**Table 2.** Influence of psychological distress trajectories on the relative risk (RR) of mental health outcomes during the COVID19 pandemic

	Psychological distress RR (s.e.), [95% CIs]			Life satisfaction RR (s.e.), [95% CIs]			Loneliness RR (s.e.), [95% CIs]		
	Wave 1	Wave 2	Wave 3	Wave 1	Wave 2	Wave 3	Wave 1	Wave 2	Wave 3
NCDS mental health trajectories Ref: No symptoms									
Stable-low symptoms	2.40 (0.32) [1.85–3.13]	1.82 (0.23) [1.43–2.32]	2.14 (0.22) [1.75–2.61]	0.94 (0.02) [0.90–0.99]	0.95 (0.02) [0.91–0.98]	0.90 (0.02) [0.86–0.94]	1.46 (0.14) [1.21–1.76]	1.50 (0.12) [1.28–1.75]	1.54 (0.08) [1.38–1.72]
Adult-onset decreasing	4.20 (0.70) [3.04–5.82]	3.26 (0.41) [2.54–4.18]	3.69 (0.37) [3.03–4.49]	0.86 (0.03) [0.80–0.92]	0.81 (0.03) [0.76–0.86]	0.82 (0.03) [0.76–0.89]	1.66 (0.19) [1.33–2.08]	1.82 (0.20) [1.47–2.25]	1.88 (0.11) [1.67–2.10]
Midlife-onset decreasing	5.80 (0.76) [4.49–7.49]	4.02 (0.48) [3.18–5.09]	4.19 (0.40) [3.46–5.07]	0.82 (0.03) [0.76–0.88]	0.78 (0.03) [0.73–0.83]	0.81 (0.03) [0.76–0.87]	2.43 (0.20) [2.07–2.85]	2.40 (0.19) [2.05–2.81]	2.15 (0.15) [1.87–2.47]
Stable-high symptoms	10.78 (1.34) [8.45–13.74]	8.28 (0.93) [6.64–10.32]	8.57 (0.71) [7.27–10.10]	0.60 (0.03) [0.54–0.68]	0.61 (0.03) [0.56–0.67]	0.61 (0.02) [0.56–0.66]	3.21 (0.22) [2.80–3.68]	3.24 (0.29) [2.71–3.86]	2.85 (0.15) [2.56–3.17]
*N*: 15 291									
BCS70 mental health trajectories Ref: no symptoms									
Early adult-onset decreasing	3.28 (0.46) [2.48–4.34]	3.08 (0.39) [2.38–3.97]	2.67 (0.28) [2.17–3.29]	0.85 (0.04) [0.77–0.94]	0.84 (0.02) [0.80–0.89]	0.85 (0.03) [0.78–0.91]	1.94 (0.19) [1.60–2.36]	1.77 (0.15) [1.48–2.10]	1.78 (0.13) [1.54–2.06]
Adult-onset decreasing	3.90 (0.62) [2.82–5.39]	3.73 (0.37) [3.07–4.54]	3.15 (0.32) [2.57–3.86]	0.82 (0.04) [0.75–0.90]	0.77 (0.03) [0.70–0.84]	0.79 (0.03) [0.73–0.87]	2.00 (0.19) [1.65–2.42]	1.94 (0.16) [1.65–2.29]	1.89 (0.15) [1.62–2.21]
Midlife-onset increasing	7.67 (0.86) [6.11–9.63]	6.66 (0.63) [5.49–8.01]	5.57 (0.49) [4.67–6.65]	0.67 (0.03) [0.61–0.74]	0.63 (0.03) [0.57–0.70]	0.69 (0.03) [0.63–0.76]	2.38 (0.22) [1.97–2.88]	2.40 (0.18) [2.06–2.79]	2.14 (0.14) [1.87–2.44]
Stable-high symptoms	10.79 (1.14) [8.71-13.36]	9.31 (0.85) [7.74–11.21]	7.56 (0.59) [6.46–8.84]	0.49 (0.04) [0.42–0.57]	0.41 (0.03) [0.36–0.47]	0.49 (0.04) [0.42–0.58]	3.09 (0.26) [2.61–3.67]	3.43 (0.21) [3.03–3.89]	3.07 (0.17) [2.75–3.42]
*N*: 16 128									

Reported as relative risk (RR), standard error (s.e.), 95% confidence intervals (95% CIs).

Outcomes are psychological distress ≥4 or not, life satisfaction ≥7 or not, loneliness ≥6 or not.

Parameters are adjusted for sex, breastfed, mother smoked during pregnancy, gestation period, birthweight, parental social class at 0, parental education at 0, housing tenure at 7/5, access to house amenities at 7/5, crowding at age 0, 7/5 and 11/10, parents marital status at 0, maternal age at birth, mother worked in first 5 years, separated from child for more than a month <age 5, read to at 7/5, CM wet the bed at 7/5, had any medical conditions at 7/5, body mass index (BMI) at 11/10, and cognitive ability at 7/5 and 11/10.

Wave 1 (May 2020), Wave 2 (September-October 2020) and Wave 3 (February–March 2021).

#### Psychological distress

In both cohorts, any pre-pandemic experience of psychological distress across the ‘adult’ life-course irrespective of age of onset, severity, longevity and proximal occurrence was associated with greater relative risk of high psychological distress during the pandemic. Mental health classes associated with the greatest risk of high psychological distress during the pandemic had more than one prior episode of poor mental health and more recent occurrences; for example during the first lockdown ‘stable-high symptoms’ were associated with a 10.8-fold increased risk in the NCDS (95% CI 8.5–13.7) and BCS70 (95% CI 8.7–13.4), and the ‘midlife-onset increasing’ trajectory in the BCS70 was related to a 7.7-fold (95% CI 6.1–9.6) increase of high psychological distress. Furthermore, ‘early adult-onset decreasing’ (mid 20s) in the BCS70 was associated with more than 3 (95% CI 2.5–4.3) times the risk, and ‘adult-onset decreasing’ (early 30s) in the BCS70 was 3.9 (95% CI 2.8–5.4) and in the NCDS 4.2 (95% CI 3.0–5.8) times the risk of high psychological distress, despite these trajectories having more favourable mental health outcomes prior to the outbreak of COVID-19.

#### Life satisfaction

In both cohorts all psychological distress trajectories (compared to ‘no symptoms’) were associated with a reduction in life satisfaction during the pandemic. In the NCDS, the risk for a reduction in life satisfaction for the ‘stable-high symptoms’ class was 40% (RR = 0.60, 95% CI 0.54–0.68) in wave 1, while for the BCS70 the risk was associated with a 51% (RR = 0.49, 95% CI 0.42–0.57) reduction in life satisfaction during the first lockdown. In addition, in the BCS70 the likelihood of high life satisfaction during the pandemic was lowered by a third (RR = 0.67, 95% CI 0.61–0.74) for the ‘midlife-onset increasing symptoms’ class.

#### Loneliness

In both cohorts all trajectories of prior psychological distress (compared with ‘no symptoms’) were associated with an increased relative risk of feelings of loneliness. In particular, ‘stable-high symptoms’ were associated with over a threefold risk (NCDS RR = 3.2, 95% CI 2.8–3.7; BCS70 RR = 3.1, 95% CI 2.6–3.7) of loneliness in both cohorts. Also, ‘midlife-onset decreasing’ in the NCDS (RR = 2.4, 95% CI 2.1–2.9) and ‘midlife-onset increasing’ in the BCS70 (RR = 2.4, 95% CI 2.0–2.9) were related with at least a doubling of the risk of feelings of loneliness.

The magnitude of the relative risk of mental health outcomes associated with each trajectory of psychological distress was similar within cohorts at different stages.

### Life-course psychological distress and mental health outcomes at different stages of the pandemic

**Figs. 3.–8.**
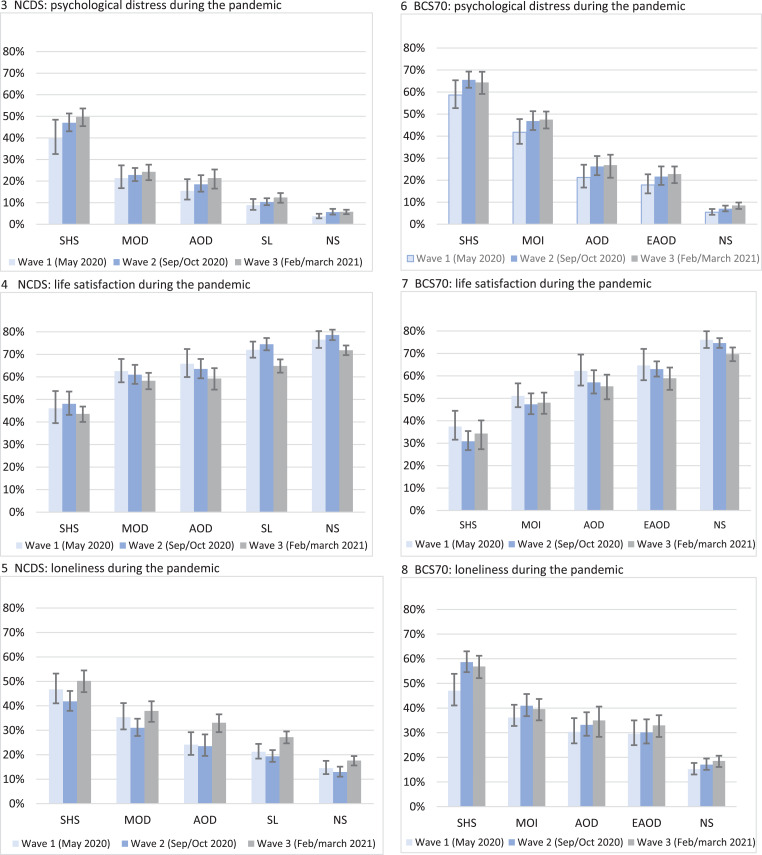
Predicted probability of mental health outcomes associated with psychological distress trajectories in the NCDS and BCS70 at different time-points during the pandemic. NS, no symptoms; SL, stable-low symptoms; EAOD, early adult-onset decreasing; AOD, adult-onset decreasing; MOD, midlife-onset decreasing; MOI, midlife-onset increasing; SHS, stable-high symptoms. Outcomes are psychological distress ≥4 or not. Parameters are adjusted for sex, breastfed, mother smoked during pregnancy, gestation period, birthweight, parental social class at 0, parental education at 0, housing tenure at 7/5, access to house amenities at 7/5, crowding at age 0, 7/5 and 11/10, parents marital status at 0, maternal age at birth, mother worked in first 5 years, separated from child for more than a month <age 5, read to at 7/5, CM wet the bed at 7/5, had any medical conditions at 7/5, body mass index (BMI) at 11/10, and cognitive ability at 7/5 and 11/10.

Figures 3–8 present the predicted probability of high psychological distress, life satisfaction and loneliness associated with psychological distress trajectories in the two cohorts at three time-points during the pandemic (predicted probabilities, standard errors and 95% confidence intervals available in online Supplementary Table 8). In the NCDS (Figs 3 and 4), psychological distress and life satisfaction were fairly constant within each of the psychological distress trajectories from May 2020 to February/March 2021. Although for the ‘stable-low’ symptoms group life satisfaction decreased from May 2020 (72.0%, 95% CI 68.5–75.7) to March 2021 (64.9%, 95% CI 62.0–67.9), and loneliness increased (W1: 21.2%, CI 18.4–24.4; W3: 27.2%, CI 24.9–29.7). Similarly, for the ‘no symptoms’ trajectory from September/October 2020 (when lockdown restrictions were relaxed), compared to February/March 2021 when the third lockdown was enforced life satisfaction decreased (W2: 78.6%, CI 76.3–81.0; W3: 71.8%, CI 69.7–74.0), and loneliness increased (W2: 12.9%, CI 11.0–15.1; W3: 17.6%, CI 15.8–19.6). Also (Fig. 5), during this period of escalating restrictions (a third lockdown) for most trajectories (‘stable-low’: W2: 19.3%, CI 17.1–21.9; W3: 27.2%, CI 24.9–29.7; ‘adult-onset’ W2: 23.5%, CI 19.5–28.3; W3: 33.1%, CI 29.6–36.9) there was an increase (‘midlife-onset’: W2: 31.0%, CI 27.7–34.7; W3: 37.9%, CI 33.9–42.3 and ‘stable-high’: W2: 41.8%, CI 38.0–46.1; W3: 50.2%, CI 46.0–54.9) in the predicted probability of loneliness.

In the BCS70 (Figs 6–8), for all the trajectories of psychological distress (with a few exceptions), the probability of worsening mental health outcomes during the pandemic was fairly constant across the three time-points. Although, for the ‘no symptoms’ group psychological distress increased (W1: 5.4%, CI 4.3–6.9; W3: 8.7%, CI 7.2–10.1) and life satisfaction decreased by March 2021 (W1: 76.1%, CI 72.4–79.9; W2: 74.1%, CI 72.4–76.8; W3: 68.8%, CI 66.7–72.8), while feelings of loneliness increased for the ‘stable-high’ symptoms trajectory from the start of the pandemic (W1: 48.4%, CI 41.1–53.9) to Autumn 2020 and Spring 2021 (W2: 59.2%, CI 54.6–63.0; W3: 58.3%, CI 52.5–61.6).

### Life course psychological distress trajectories and mental health outcomes after controlling for midlife factors

**Figs. 9–14.**
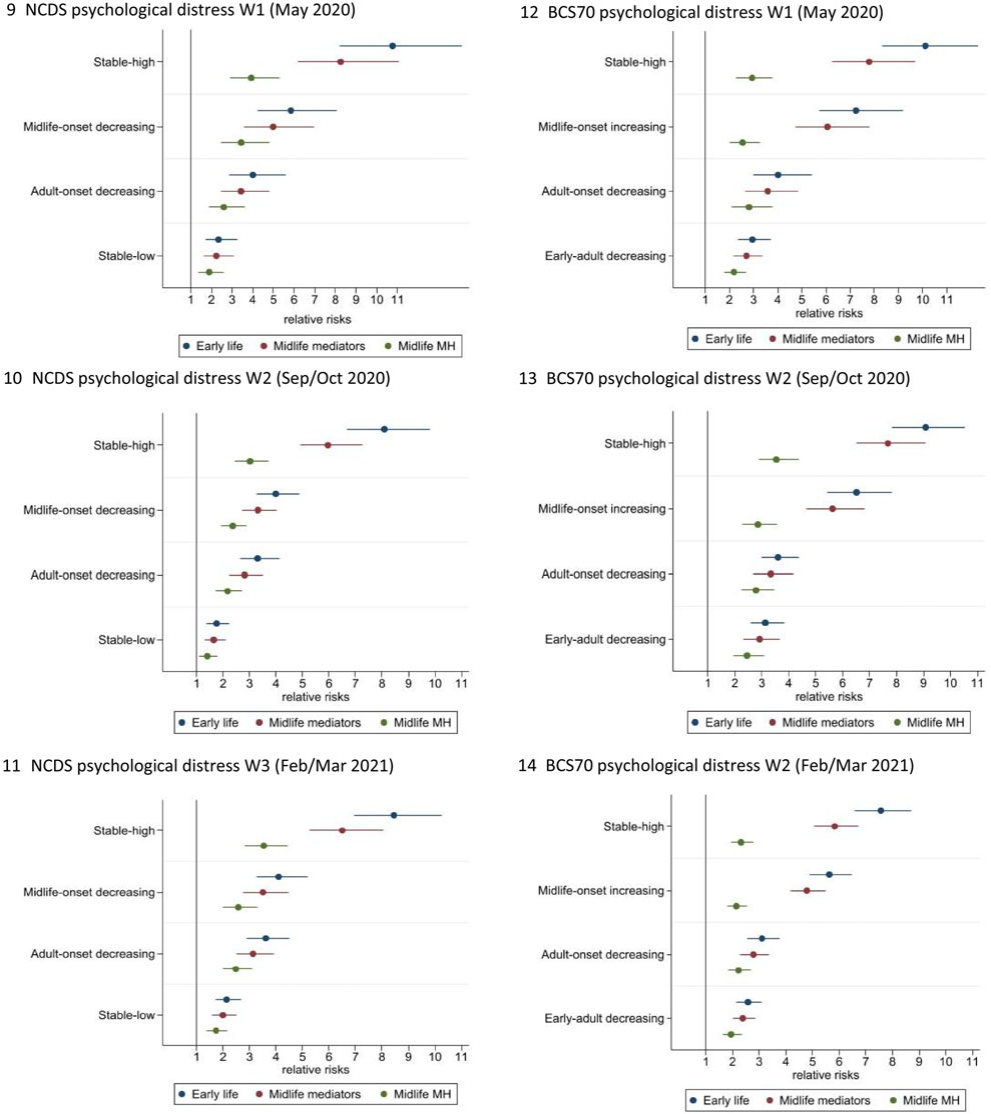
Relative risk of psychological distress during the pandemic associated with pre-pandemic psychological distress groups (compared to ‘no symptoms’) after controlling for early-life factors, midlife mediators, and midlife mediators and mental health in midlife. Reported as relative risk (RR) and 95% confidence intervals (95% CIs). Outcomes are psychological distress ≥4 or not. Wave 1 (May 2020), Wave 2 (September to October 2020) and Wave 3 (February–March 2021). Parameters: all models are adjusted for sex, breastfed, mother smoked during pregnancy, gestation period, birthweight, parental social class at 0, parental education at 0, housing tenure at 7/5, access to house amenities at 7/5, crowding at age 0, 7/5 and 11/10, parents marital status at 0, maternal age at birth, mother worked in first 5 years, separated from child for more than a month <age 5, read to at 7/5, CM wet the bed at 7/5, had any medical conditions at 7/5, body mass index (BMI) at 11/10, and cognitive ability at 7/5 and 11/10. Midlife mediators adjusted for social class, highest educational attainment, income, employment status, housing tenure, cognitive function, general health, smoked, drinking, BMI, household size, number of children and partnership status. Midlife mediators and mental health: psychological distress at age 50 in the NCDS and age 46 in BCS70.

Figures 9–14 present the relative risks in the fully adjusted models, associated with psychological distress during the pandemic for different trajectories of mental health presented in Table 2, alongside models including socio-economic, health and cognitive midlife explanatory variables, and additional models also including psychological distress at age 50 and 46 respectively in the NCDS and BCS70, to investigate if life-course psychological distress groupings explain mental health outcomes over and above these factors. Tables reporting the relative risk (RR), standard errors and 95% confidence intervals for all mental health outcomes are available in online Supplementary Tables S9.1 and S9.2.

In both cohorts controlling for midlife factors and in particular most recent pre-pandemic psychological distress explained much, but not all, of the association between pre-pandemic life-course psychological distress trajectories and mental health during the pandemic. In the NCDS, compared to the ‘no-symptoms’ group just under two-thirds (58.8–63.6%, dependent on time during the pandemic) of the relative risk of psychological distress was explained in the ‘stable-high’ group, 38.7–41.3% in the ‘midlife-onset decreasing’ group, around a third (32.8–38.1%) in the ‘adult-onset’ and a fifth (19.2–23.1%) in the ‘stable-low’ symptoms group. Likewise, in the BCS70 midlife factors explained a high proportion (between 63.3% and 72.8%, wave dependent) of the relative risk of psychological distress during the pandemic for the ‘stable-high’ symptoms group, 57.5–66.6% for the ‘midlife-onset increasing’, 25.7–29.2% for the ‘adult-onset’ and 19.5–33% for the ‘early adult-onset’ group. However, even after controlling for early life and midlife factors including the most recent pre-pandemic measure of psychological distress, the relative risks associated with pre-pandemic life-course psychological distress trajectories and poor mental health during the pandemic were sizeable. The ‘stable-high’ symptoms group was associated with a 3.0/3.9-fold increased risk in the NCDS (95% CI 2.4/2.9–3.7/5.3) and 2.3–3.4 times the risk in the BCS70 (95% CI 1.9/2.9–2.5/3.5), and also the ‘midlife-onset’ trajectory in the BCS70 was related to a 2.1/2.6 (95% CI 1.8/2.3–2.6/3.5) and in the NCDS a 2.4/3.4 (95% CI 1.9/2.5–2.9/4.8) risk in poor mental health during the pandemic. Furthermore, all other trajectories compared to the ‘no-symptoms’ group were associated with a greater risk of poor mental health during the pandemic; ‘adult-onset’ in the NCDS were at a 2.2/2.6 (95% CI 1.7/2.0–2.7/3.6) and in the BCS70 a 2.2/2.8 (95% CI 1.8/2.3–2.7/3.8) fold increased risk, ‘early adult-onset decreasing’ in the BCS70 was associated with a 1.9/2.5 (95% CI 1.6/2.0–2.3/3.0), and the ‘stable-low’ symptoms group in the NCDS 1.4/1.9 (95% CI 1.1/1.4–1.8/2.6) times the risk of psychological distress during COVID-19.

## Discussion

We identified five distinct trajectories of psychological distress from adolescence to midlife. All trajectories with prior symptoms of psychological distress across the life-course irrespective of age of onset, severity, longevity and proximal occurrence were associated with a greater relative risk of high psychological distress, reduction in life satisfaction, as well as feelings of loneliness during the pandemic. Pre-pandemic trajectories featuring more than one prior episode of poor mental health and more recent occurrences predicted the greatest risk of mental distress during the pandemic. The probability of high psychological distress, and lower life satisfaction (and loneliness for the BCS70) associated with psychological distress trajectories in the two cohorts remained fairly stable at three time-points during the pandemic, while in the NCDS, feelings of loneliness increased for most trajectories during the third lockdown when compared to early Autumn when restrictions had been relaxed and for the ‘stable-high’ trajectory in the BCS70 from May 2020 as the pandemic progressed. Accounting for midlife mediators, including most recent pre-pandemic psychological distress not surprisingly explained a large proportion (from a fifth to two-thirds in the NCDS and a fifth to three-quarters in the BCS70 dependent on trajectory and timing during the pandemic) of the relationship between pre-pandemic life-course psychological distress trajectories and mental health during the pandemic. However, the relationship remained sizeable, the relative risk of poor mental health during the pandemic for pre-pandemic psychological distress trajectories, compared to no-symptoms ranged from 1.9 to 3.9 in the NCDS and 2.5 to 3.4 in the BCS70 suggesting these groups are less resilient to shocks, such as pandemics.

The pre-pandemic trajectories identified here are similar to the age of onset and recurrence of three of six classes and three of three classes described in prior studies covering equivalent age periods (Colman et al., 2007; Paksarian et al., 2016). The latter study acknowledging, with only three classes the sample sizes reduced the complexity of the models that could be estimated. This study identified five trajectories in each cohort, with slight observed differences in trajectories across generations, which may be cohort effects; for example the period 1990–1993 was marked by a long recession when the BCS70 cohort members would have been in their early 20s and new entrants to the labour market (Sullivan, Brown, & Bann, 2015), perhaps resulting in increased levels of distress for some during this period. Nevertheless, the age of onset found in these mental health trajectories are in line with age of onset for generalised anxiety disorder (median 24–50) and mood disorders (median 29–40) worldwide (Kessler et al., 2007). In this study, although the most recent pre-pandemic measure of psychological distress explained much of the relationship between the trajectories and mental health during the pandemic, the relation between adult life-course trajectories of psychological distress and poor mental health during the pandemic remained important, suggesting prior poor mental health during the adult life-course, and not just most recent measures of psychological distress are notable in identifying individuals less resilient to shocks, such as pandemics.

This study highlights the potential danger of dividing the population into symptomatic and asymptomatic groups. As other studies have illustrated symptoms are dimensional and can range from none to severe. In particular, the future mental health risks faced by those with subthreshold depression and anxiety have been shown to be associated with previous and future severe disorder (Bosman et al., 2019; Fergusson, Horwood, Ridder & Beautrais, 2005). In addition, when comparing different pre-pandemic trajectories of psychological distress (online Supplementary Tables S11.1 and S11.2), there were distinct relative risks associated with different mental health trajectories and mental health outcomes for most comparisons during the pandemic, thus illustrating the associated influence of heterogeneity of life-course mental health on future outcomes. Moreover, assuming mental well-being at one time point is indicative of an individuals' predisposition to future mental health outcomes could underestimate the importance of life-course mental health for mental health outcomes during the pandemic (Kessler et al., 2005; Moffitt et al., 2010). Population studies, focusing on depression have reported around 40–50% of those who recovered experienced a recurrence (Mattisson, Bogren, Horstmann, Munk-Jorgensen, & Nettelbladt, 2007), as well as comorbidity and transitions between disorders over time (Merikangas et al., 2003). In our study, the associated risk of poor mental health outcomes during the pandemic was greater for trajectories of psychological distress with recent occurrences, and with more than one prior episode. Indeed, those with prior mental health problems have been found to be at greater risk of post disaster mental health illness (Goldmann & Galea, 2014).

### Strengths and limitations

Our study is the first to relate longitudinal mental health data across more than 30 years before the COVID-19 pandemic within the same individuals to their mental health outcomes during the COVID-19 pandemic. In addition, we identify trajectories of individuals with differing experience of psychological distress over time during adulthood in two cohorts, rather than treating longitudinal trajectories of mental health as homogenous. Other unique strengths include nationally representative samples, the sample size and prospective follow-up hitherto from birth to midlife, together with data collected at three discrete time points during the COVID-19 pandemic from May 2020 to March 2021.

There are a number of limitations of this study which are important to note. Our findings can only be generalised to those born in Britain in 1958 and 1970 or close to those years. Furthermore, our data are derived from two observational longitudinal studies and there may be bias due to unmeasured confounding. However, sensitivity analyses with the *E* value (VanderWeele & Ding, 2017) suggest that for the observed associations with pre-pandemic life-course psychological distress trajectories and mental health during the pandemic, strong confounding, stronger than that observed in our data, would be needed to completely explain away our findings (online Supplementary Table S10). Also with most longitudinal research, there was an issue of selective attrition. However, we capitalised on the rich information available in both cohorts augmenting our models with auxiliary variables in the multiple imputation models, including variables related to non-response to the COVID-19 surveys, mental health and related variables from birth. This approach allows for predicting missing data with greater accuracy and minimizing non-random variation in these values (Sterne et al., 2009), improves the plausibility of the missing-at-random assumption and restores sample representativeness (Mostafa et al., 2021). However, bias due to a non-ignorable missing data generating mechanism cannot be ruled out. The study includes extensive data on pre-pandemic mental health, social, economic and demographic characteristics. However, as we have no counterfactual at the time of the pandemic, we cannot assume the relative risks of poorer mental health outcomes were directly the result of the pandemic itself, ensuing circumstances and/or other factors. As with most research conducted during the pandemic, interviews were conducted online, and may have resulted in measurement bias and potential mode effects (Goodman et al., 2022), we cannot preclude that differences in results may be due to ‘true’ differences, measurement error or a mode effect (or a combination of those). However, evidence shows measurement invariance between all pre-pandemic and COVID-19 measurements of psychological distress in both cohorts suggesting mode effects did not bias our findings (Moreno-Agostino et al., 2022). In terms of the method adopted, latent classes are approximations of symptom patterns in the data and do not represent actual data points, but are evidenced-based summaries of mental health in the cohorts. In addition to this, with respect to the pre-COVID measures, we only had five ages where psychological distress was measured, and the most recent assessment was 4 and 12 years prior to the COVID-19 pandemic in the BCS70 and NCDS respectively. And, although we identified five distinct trajectories, which were similar, but not identical in two cohorts, we can estimate the association within cohort but cannot directly compare specific trajectories across cohorts and their association with mental health during the pandemic. Furthermore, for both studies we used different measures and reporters (the parent at age 16 and self-report in adulthood) of psychological distress at age 16, compared to adulthood. We acknowledge that correlations between parent and self-report tend to be low (Collishaw, Goodman, Ford, Rabe-Hesketh, & Pickles, 2009). As self-report Malaise Inventory was available at age 16 in the BCS70 only, we reran the models and identified the same trajectories for the five classes identified using the original measures (online Supplementary Fig. S1) and a difference in trajectory classification of only 2.9% (online Supplementary Table S12).

In conclusion, data from two British birth cohorts suggest that for different life-course trajectories of psychological distress there were distinct relative risks of poor mental health outcomes during the pandemic. Whilst any prior symptoms of psychological distress regardless of age of onset, severity and chronicity put individuals at greater risk, those with chronic and more recent occurrences were likely to require more support. We show fairly stable mental health outcomes during the pandemic for distinct life-course mental health trajectories. Although, midlife factors especially the most recent pre-pandemic psychological distress explained much of the relationship between pre-pandemic life-course psychological distress trajectories and mental health during the pandemic, the relation between life-course psychological distress and mental health during the pandemic remained strong, suggesting these groups might be more susceptible to shocks. Our findings show the importance of considering heterogeneous mental health trajectories across the life-course in the general population in addition to mental health average population trends.

## Data Availability

The data are freely available from the UK Data Service at https://ukdataservice.ac.uk/.

## Supplementary material

For supplementary material accompanying this paper visit https://doi.org/10.1017/S0033291722003877. click here to view supplementary material
