# Supplementary material for: Association between psychological distress trajectories from adolescence to midlife and mental health during the pandemic: evidence from two British birth cohorts
Source: Psychol Med. 2023 Mar 20;53(14):6547–59. doi: 10.1017/S0033291722003877 (PMC10600943; doi:10.1017/S0033291722003877)
Supplement: Supplementary file 1 [file S0033291722003877sup001.docx]

# Supplementary appendix

**Table S1: Sample distribution of cohort members across all covariates by cohort**

|  | **NCDS**  **%/mean** | **BCS70**  **%/mean** |
| --- | --- | --- |
| **Mental health trajectory** |  |  |
| No symptoms | 40.9 | 52.8 |
| Stable-low/Early adult-onset decreasing | 16.4 | 9.1 |
| Adult-onset decreasing | 10.8 | 11.0 |
| Midlife-onset decreasing / Midlife-onset increasing | 11.9 | 7.8 |
| Stable-high symptoms | 19.9 | 19.3 |
| **Early life factors** |  |  |
| **Sex** |  |  |
| Male | 50.6 | 51.1 |
| Female | 49.4 | 48.9 |
| **Breast Fed** |  |  |
| Yes | 67.9 | 36.7 |
| No | 32.1 | 63.3 |
| **Smoked during pregnancy** |  |  |
| Yes | 29.4 | 41.0 |
| No | 70.6 | 59.0 |
| **Gestation period (days)** | 280.8 | 281.3 |
| **Birthweight (grams)** | 3329.9 | 3302 |
| **Family characteristics** |  |  |
| **Parental social class (RGSC) (0)** |  |  |
| Professional | 4.6 | 4.3 |
| Managerial | 12.9 | 22.0 |
| Skilled non-manual | 10.1 | 10.0 |
| Skilled manual | 50.0 | 38.5 |
| Semi-skilled manual | 12.7 | 13.7 |
| Unskilled manual | 9.8 | 6.8 |
| Retired/unemployed | - | 4.7 |
| **Fathers education (post compulsory age)** |  |  |
| Yes | 23.0 | 19.9 |
| No | 77.0 | 80.1 |
| **Mothers education (post compulsory age)** |  |  |
| Yes | 25.0 | 17.7 |
| No | 75.0 | 82.3 |
| **Housing Tenure (5)** |  |  |
| Home owner | 42.0 | 55.3 |
| Other | 58.0 | 44.7 |
| **Total household income** |  |  |
| 1 Lowest quintile | 23.3 | 26.9 |
| 2 | 20.2 | 21.4 |
| 3 | 19.6 | 19.1 |
| 4 | 18.9 | 16.9 |
| Highest quintile | 18.0 | 15.7 |
| **Access to house amenities** |  |  |
| Sole | 80.8 | 94.0 |
| Not sole | 19.2 | 6.0 |
| **Crowding (age 7/5)** |  |  |
| < 1 person per room | 57.1 | 35.9 |
| 1 to 1.49 person per room | 27.7 | 51.2 |
| 1.5 or more person per room | 15.3 | 12.9 |
| **Crowding (age 11/10)** |  |  |
| < 1 person per room | 60.5 | 30.3 |
| 1 to 1.49 person per room | 27.2 | 52.3 |
| 1.5 or more person per room | 12.3 | 17.4 |
| **Marital status** |  |  |
| Married | 96.1 | 92.4 |
| Other | 3.9 | 7.6 |
| **Maternal age at birth** | 27.5 | 25.9 |
| **Mother worked in first 5 years** |  |  |
| Yes | 29.4 | 30.9 |
| No | 70.6 | 69.1 |
| **Separated from child >1 month** |  |  |
| Yes | 12.0 | 5.6 |
| No | 88.0 | 94.4 |
| **Child health** |  |  |
| **Wet the bed at age 5** |  |  |
| Yes | 10.8 | 22.5 |
| No | 89.2 | 77.5 |
| **Had any medical conditions** |  |  |
| None | 56.6 | 46.4 |
| One | 27.7 | 29.9 |
| Two or more | 15.6 | 23.7 |
| **Adult social class**  Professional and Managerial  Intermediate  Routine and manual  Never worked | 35.7  26.9  31.5  5.9 | 36.4  18.7  24.4  20.4 |
| Highest adult education  Degree | 18.4 | 18.0 |
| Income  1 Lowest quintile  2  3  4  Highest quintile | 20.6  21.0  20.4  18.4  19.6 | 25.8  23.0  17.6  17.1  16.5 |
| Housing tenure  Own home | 84.8 | 65.0 |
| Household size | 2.6 | 3.1 |
| Number of children | 2.1 | 1.9 |
| Partnership status  Co-habiting | 76.6 | 69.7 |
| Cognitive function | -.0.09 | -0.14 |
| General health  Poor  Fair  Good  Very good  Excellent | 7.4  16.4  32.9  31.4  11.8 | 8.4  17.4  29.5  30.5  14.2 |
| Ever smoked  Currently  Occasionally  Used to  Never | 16.2  4.1  32.1  47.6 | 20.8  6.7  31.5  40.9 |
| Problem drinking | 17.3 | 29.0 |
| Adult BMI | 27.50 | 28.0 |
| Adult mental health | 1.68 | 2.02 |

**Table S2: Response by sweep**

|  | **NCDS** | **BCS70** |
| --- | --- | --- |
| **Birth** | 17,415 | 17,196 |
| Age 7/5 | 15,425 | 13,135 |
| Age 11/10 | 15,337 | 14,875 |
| Age 16 | 14,654 | 11,622 |
| Age 23/26 | 12,537 | 9,003 |
| Age 33/34 | 11,469 | 9,665 |
| Age 42 /42 | 11,419 | 9,841 |
| Age 50 /46 | 9,790 | 8,581 |
| Age 55 | 9,137 | - |
| **COVID-19 Sweeps** |  |  |
| Wave 1 | 5,178 | 4,223 |
| Wave 2 | 6,282 | 5,320 |
| Wave 3 | 6,757 | 5,684 |

**Table S3: Influence of psychological distress trajectories on mental health outcomes during the COVID19 pandemic**

**(Impute and delete’ method^1^).**

|  | Psychological distress  RR, 95% CIs | | | Life satisfaction  RR, 95% CIs | | | Loneliness  RR, 95% CIs | | |
| --- | --- | --- | --- | --- | --- | --- | --- | --- | --- |
|  | Wave 1 | Wave 2 | Wave 3 | Wave 1 | Wave 2 | Wave 3 | Wave 1 | Wave 2 | Wave 3 |
| NCDS mental health trajectories  Ref: No symptoms |  |  |  |  |  |  |  |  |  |
| Stable-low symptoms | 1.95 (0.52)  [1.16, 3.29] | 1.79 (0.44)  [1.11, 2.90] | 2.35 (0.46)  [1.60, 3.47] | 0.92 (0.03)  [0.86, 0.98] | 0.95 (0.04)  [0.88, 1.02] | 0.90 (0.04)  [0.83, 0.97] | 1.46 (0.22)  [1.09, 1.97] | 1.44 (0.24)  [1.04, 1.99] | 1.63 (0.19)  [1.29, 2.05] |
| Adult-onset decreasing | 3.67 (0.99)  [2.15, 6.22] | 3.17 (0.89)  [1.83, 5.51] | 3.24 (0.75)  [2.05, 5.13] | 0.86 (0.05)  [0.77, 0.97] | 0.84 (0.06)  [0.73, 0.96] | 0.85 (0.06)  [0.74, 0.97] | 1.59 (0.33)  [1.06, 2.39] | 1.85 (0.35)  [1.28, 2.70] | 1.82 (0.32)  [1.29, 2.56] |
| Midlife-onset decreasing | 5.08 (1.30)  [3.07, 8.41] | 3.46 (0.84)  [2.14, 5.60] | 3.72 (0.74)  [2.52, 5.49] | 0.85 (0.04)  [0.77, 0.93] | 0.79 (0.06)  [0.68, 0.91] | 0.87 (0.04)  [0.79, 0.97] | 2.35 (0.35)  [1.75, 3.17] | 2.56 (0.40)  [1.88, 3.49] | 2.09 (0.27)  [162, 2.70] |
| Stable-high symptoms | 9.36 (2.10)  [6.01, 14.56] | 6.53 (1.43)  [4.24, 10.06] | 7.73 (1.29)  [5.57, 10.72] | 0.65 (0.05)  [0.56, 0.75] | 0.70 (0.06)  [0.60, 0.81] | 0.67 (0.05)  [0.57, 0.77] | 2.98 (0.43)  [2.24, 3.95] | 2.79 (0.43)  [2.04, 3.78] | 2.87 (0.34)  [2.28, 3,62] |
| N: | 4,727 | 6,067 | 6,539 | 4,812 | 6,228 | 6,580 | 4,748 | 6,078 | 6,566 |
| BCS70 mental health trajectories  Ref: No symptoms |  |  |  |  |  |  |  |  |  |
| Early adult- onset decreasing | 2.78 (0.43)  [2.05, 3.76] | 2.98 (0.32)  [2.41, 3.68] | 2.77 (0.29)  [2.25, 3.41] | 0.87 (0.03)  [0.83, 0.94] | 0.85 (0.03)  [0.80, 0.90] | 0.85 (0.03)  [0.79, 0.91] | 1.87 (0.19)  [1.53, 2.28] | 1.89 (0.16)  [1.59, 2.23] | 1.82 (0.13)  [1.57, 2.10] |
| Adult-onset decreasing | 3.86 (0.56)  [2.90, 5.14] | 3.66 (0.41)  [2.93, 4.56] | 3.27 (0.35)  [2.65, 4.02] | 0.84 (0.03)  [0.67, 0.80] | 0.81 (0.03)  [0.76, 0.87] | 0.81 (0.03)  [0.75, 0.88] | 2.02 (0.21)  [1.64, 2.49] | 1.98 (0.18)  [1.65, 2.37] | 1.88 (0.15)  [1.61, 2.19] |
| Midlife- onset decreasing | 7.22 (0.84)  [5.74, 9.07] | 6.27 (0.55)  [5.27, 7.46] | 5.71 (0.49)  [4.83, 6.76] | 0.73 (0.03)  [0.70, 0.80] | 0.67 (0.03)  [0.61, 0.73] | 0.72 (0.03)  [0.66, 0.78] | 2.40 (0.22)  [2.01, 2.88] | 2.58 (0.20)  [2.22, 3.00] | 2.13 (0.15)  [1.61, 2.19] |
| Stable-high symptoms | 10.60 (1.13)  [8.60, 13.06] | 9.12 (0.73)  [7.82, 10.68] | 7.87 (0.61)  [6.77, 9.15] | 0.59 (0.03)  [0.52, 0.65] | 0.51 (0.03)  [0.46, 0.57] | 0.56 (0.03)  [0.50, 0.62] | 3.11 (0.26)  [2.65, 3.66] | 3.68 (0.24)  [3.24, 4.18] | 2.98 (0.18)  [2.64, 3.35] |
| N: | 3,768 | 4,870 | 5,291 | 3,805 | 4,888 | 5,311 | 3,776 | 4,885 | 5,303 |

Reported as Relative Risk (RR), 95% confidence intervals (CIs)

Parameters are adjusted for sex, breastfed, mother smoked during pregnancy, gestation period, birthweight, parental social class at 0, parental education at 0, housing tenure at 7/5, access to house amenities at 7/5, crowding at age 0, 7/5 and 11/10, parents marital status at 0, maternal age at birth, mother worked in first five years, separated from child for more than a month <age 5, read to at 7/5, CM wet the bed at 7/5, had any medical conditions at 7/5, Body Mass Index (BMI) at 11/10, and cognitive ability at 7/5 and 11/10.

Wave 1 (May 2020), Wave 2 (September to October 2020) and Wave 3 (February-March 2021)

**Table S4: Details of covariates**

| Variable | Age of cohort member (CM) | Description |
| --- | --- | --- |
| Sex | 0 | Male=0 (ref) Female =1 |
| Maternal age at birth | 0 | Maternal age at birth in years |
| Breast-fed or not | 0 | Whether the mother ever breastfed the infant (1) or not (0) |
| Smoked during pregnancy | 0 | Whether during pregnancy the mother smoked more than 1 cigarette daily (1), or not (0) |
| Gestation period | 0 | Gestation period in days |
| Birthweight | 0 | Birthweight recorded in grams |
| Parental social class | 0 | Occupation of the father was coded according to the Registrar General’s classification. Participant’s current or most recent jobs were classified as: 1 professional, 2 managerial and technical, 3 skilled non-manual 4 skilled manual, 5 partly-skilled manual, and 6 unskilled. |
| Father’s education | 0 | Father stayed on at school after the minimum school leaving age, yes (1) no (0). |
| Mother’s education | 0 | Mother stayed on at school after the minimum school leaving age, yes (1) no (0). |
| House tenure | 7 in NCDS and 5 in BCS70 | Whether the CM’s parents were home owners (1), or not (0). |
| Access to house amenities | 7 in NCDS and 5 in BCS70 | Whether the family had sole access to the following household amenities; bathroom, indoor WC, hot water, and kitchen (1) or not (0) |
| Crowding | 0, 7, 11 in NCDS and 0, 5, 10 in BCS70 | Number of persons per room (excluding kitchen, toilet, and bathroom), up to 1 (0), 1 to 1.49 (1) and 1.5 or over (2). |
| Marital status | 0 | Whether parents were married (1) or not (0). |
| Read to child every week | 7 in NCDS and 5 in BCS70 | CM read to at least once a week, yes (1) no (0). |
| Mother worked before CM went to school | 7 in NCDS and 5 in BCS70 | Whether the mother worked full-time or part time before the child went to school, yes (1) no (0). (In the NCDS, mothers were asked whether they had paid work outside the home since CM’s birth and before child started school. In the BCS70, (at age 5) the mother was asked if they had a regular full-time or part-time job out of the home since the time of CM’s birth). |
| Separated with CM for more than one month | 7 in NCDS and 5 in BCS70 | Whether the mother has been separated from CM for one month or more, yes (1) no (0). |
| Bedwetting | 7 in NCDS58 and 5 in BCS70 | In the NCDS, whether CM has wet the bed since age 5, yes (1) no (0). In the BCS70, at age 5, does the CM wet the bed, yes (1) no (0). |
| Medical conditions | 7 in NCDS and 5 in BCS70 | Whether the CM had any of the following medical conditions; eczema, hay fever and sneezing, ear discharge, sore throats, bronchitis, pneumonia, hearing difficulty. The medical conditions were recoded into, none (0), one (1), two or more (2). |
| BMI | 11 in NCDS and 10 in BCS70 | Height and weight were measured by trained medical personnel using standard protocols at age 11 in the NCDS and at age 10 in BCS70. BMI was standardised, with a mean of 0 and a standard deviation of 1. |
| Cognitive ability | 7, 11 in NCDS and 5, 10 in BCS70 | In the NCDS at age 7, CM’s completed the Southgate Group Reading Test, Problematic Arithmetic Test, Copy Design Test and Human Figure Drawing; at age 11 they completed a General Ability Test (both verbal and non-verbal), a Reading Comprehension Test, a Mathematics Test and Copy designs Test. In the BCS70 at age 5 CM’s completed the English Picture Vocabulary Test, Copy Designs Test, Human Figure Drawing and Complete a Profile Test; at age 10 CM’s completed four tests from the British Ability Scales, Word Similarities, Word definitions, Recall of Digits and Matrices.^2^ All tests were standardised, with a mean of 0 and standard deviation of 1. Within each age group and cohort, a Principal Component Analysis (PCA) was conducted to extract the common variance across all the cognitive tests and a single component predicted representing a general ability score. |
| Adult social class | Age 55 in NCDS and 46 in BCS70 | Current occupation coded according to the National Statistics Socio-economic Classification (NS-SEC). Participant’s current job was classified as: 0 Managerial / Professional, 1 Intermediate 2 Routine and manual 3 Never worked. |
| Highest adult education | Age 55 in NCDS and 46 in BCS70 | 0 No degree, 1 Degree or higher |
| Income | Age 55 in NCDS and 46 in BCS70 | Income after tax and reductions in quintiles |
| Employment status | Age 55 in NCDS and 46 in BCS70 | Current employment status 0 Full-time employment, 1 Part-time employment, 2 Self-employed, 3 Not working |
| Housing tenure | Age 55 in NCDS and 46 in BCS70 | 0 Not homeowner, 1 Homeowner |
| Household size | Age 55 in NCDS and 46 in BCS70 | Number of persons living in household |
| Number of children | Age 55 in NCDS and 46 in BCS70 | Total number of children |
| Partnership status | Age 55 in NCDS and 46 in BCS70 | Cohabiting as a couple 0 No 1 Yes |
| Cognitive function | Age 50 in NCDS and 46 in BCS70 | In mid-life CMs completed tests of cognitive function including verbal fluency, short-term memory and processing speed ^2.^ All tests were standardised, with a mean of 0 and standard deviation of 1. Within each cohort, a Principal Component Analysis (PCA) was conducted to extract the common variance across all the cognitive tests and a single component predicted representing mid-life cognitive function. |
| General health | Age 55 in NCDS and 46 in BCS70 | Self-reported 5 Excellent, 4 Very good, 3 Good, 2 Fair, 1 Poor |
| Ever smoked | Age 55 in NCDS and 46 in BCS70 | 0 Never, 1 Used to smoke, 2 Currently smoke |
| Drinking | Age 50 in NCDS and 46 in BCS70 | Using the AUDIT (Alcohol Use Disorders Identification Test) which consists of 10 questions covering alcohol consumption, problems and dependency. Responses to each question are scored from 0 to 4 giving a maximum score of 40 (nd8audit). 0 No problem drinking (Scores of <8) 1 Problem drinking (Scores of 8 or more). |
| Adult BMI | Age 55 in NCDS and 46 in BCS70 | Height and weight were measured by trained medical personnel using standard protocols. BMI was standardised, with a mean of 0 and a standard deviation of 1. |
| Adult mental health | Age 50 in NCDS and 46 in BCS70 | Psychological distress was measured in both cohorts with the nine-item version of the Malaise Inventory. The Inventory ranges from 0 to 9, a higher score identifying greater psychological distress. |

**Table S5.1 and S5.2: Details on latent class analysis**

The analytic sample for the latent class analysis (LCA) included all individuals with at least four assessments of psychological distress from adolescence to midlife, and who were alive and had not emigrated (n=11,579 in the NCDS and n=10,236 in the BCS70) by the age of 50 and 46 respectively. Missing data within the LCA was modelled with Full Information Maximum Likelihood.^3^

As would be expected in the general population, many cohort members had no or few symptoms of psychological distress. As a result, we applied a four-category ordinal variable on the basis of grouping factor scores distributions of the latent measures to each of the five time points in each cohort. The first group consisted of individuals from the 1^st^ to 50^th^ percentile on the factor scores (i.e. lack of or no symptoms), the second included individuals from the 51^st^ to the 75^th^ percentile (i.e. occasional symptoms), the third group included individuals from the 76^th^ and 90^th^ percentile (i.e. moderate symptoms), and the fourth group individuals from the 91^st^ to 100^th^ percentile (i.e. severe symptoms).

Based on previous studies, we expected the number of trajectories to range from 3 to 6 classes, one or more stable low or no symptoms group, a class with persistently high symptoms, and groups of unstable symptoms either decreasing or increasing over time or moderate stable symptoms group depending on different populations.^4^ Latent classes are represented by a categorical latent variable and are classified based on similar patterns of in this study, observed longitudinal data. Every individual in the data set has their own probabilities calculated in all of the latent classes estimated and the classes are based on these probabilities. As latent classes are unobserved and the appropriate number of latent classes initially unknown, based on prior research we explored models with three to seven classes (one more class than was expected^5^) to identify the most parsimonious model (s-Table 4.1 and 4.2).

We used several indices of goodness of fit to compare models with different number of classes; a bootstrap p value for the likelihood ratio $x^{2}$ test (p<.01 indicating good fit), Bayesian Information Criteria (lowest value has best fit), Akaike’s Information Criteria (lowest value has best fit), and the Lo-Mendell-Rubin likelihood ratio bootstrap p value to compare whether an additional latent class improves model fit. In addition, we used Entropy a composite that indicates the overall ability of a mixture model to return well-separated profiles.^7^ All Latent mixture modelling was conducted using MPLUS v8.2(Muthén and Muthén).

**Table S5.1: Indices of model fit for a Latent Profile Model in NCDS**

|  | 3 classes | 4 classes | 5 classes | 6 classes | 7 classes |
| --- | --- | --- | --- | --- | --- |
| Likelihood ratio bootstrap p valueᵅ | <.001 | <.001 | <.01 | <.001 | <.001 |
| Bayesian Information Criteriaᵇ | 126,305 | 123,017 | 121,283 | 121,313 | 120.295 |
| Akaike’s Information Criteriaᶜ | 126,144 | 122,811 | 121,033 | 121.020 | 119,957 |
| Lo-Mendell-Rubin bootstrap p value of likelihood ratio test ᵈ | <.001 | <.001 | <.001 | .427 | <.001 |
| Entropyᵉ | .830 | .836 | .903 | .813 | .810 |

ᵅ >.01 indicates good fit. ᵇlowest value indicate best fit. ᶜ lowest value indicate best fit. ᵈ comparing current to previous model, p<.05 indicates addition of this class significantly improves fit. ᵉhigher entropy (up to perfect classification at 1) indicate a better fit

**Table S5.2: Indices of model fit for a Latent Profile Model in BCS70**

|  | 3 classes | 4 classes | 5 classes | 6 classes | 7 classes |
| --- | --- | --- | --- | --- | --- |
| Likelihood ratio bootstrap p valueᵅ | <.001 | <.001 | <.001 | <.001 | <.001 |
| Bayesian Information Criteriaᵇ | 94,278 | 93,621 | 92,799 | 92,192 | 91,557 |
| Akaike’s Information Criteriaᶜ | 94,277 | 93,419 | 92,554 | 91,903 | 91,225 |
| Lo-Mendell-Rubin bootstrap p value of likelihood ratio test ᵈ | <.001 | <.001 | <.05 | <.001 | <.001 |
| Entropyᵉ | .788 | .772 | .782 | .777 | .744 |

ᵅ >.01 indicates good fit. ᵇlowest value indicate best fit. ᶜ lowest value indicate best fit. ᵈ comparing current to previous model, p<.05 indicates addition of this class significantly improves fit. ᵉhigher entropy (up to perfect classification at 1) indicate a better fit

In the NCDS, when comparing 5 to 6 classes the Lo-Mendell-Rubin bootstrap p value of likelihood ratio test indicated that the addition of another latent class did not significantly improve model fit. In addition, the entropy in the 5 class model increases to 0.903 from the 4 class model and declines again from the 5 to 6 class solution. In the BCS70, the Lo-Mendell-Rubin bootstrap p value of likelihood ratio test was significant for all classes and the entropy was similar across all models, albeit declining from class 5 0.782 to 0.744 in the 7 class model. An additional consideration is the size of the smallest class, if this is composed of a relatively small number of members, little is gained given the possibility of low power and precision relative to the other, larger classes ^7^. In the 6 class model (in the BCS70), the prevalence of the 6^th^ class was low (3.1%), along with the probability of classification to the group 0.540. In both cohorts we therefore selected the 5 class model as the most parsimonious summary of psychological distress from adolescence to midlife.

The five longitudinal groups were not identical in the two cohorts, most of the groupings were very similar. In both cohorts, the largest group had ‘no symptoms’ (NCDS n = 5057 (43.7%), BCS70 n = 5821, (56.9%), There was also a group with persistent and repeated severe symptoms ‘stable-high’ (NCDS n=1749 (15.1%), BCS70 n=1275 (12.5%), and a group with adult-onset and decreasing symptoms ‘adult-onset decreasing’ (NCDS=1172 (10.1%), BCS70=1065(10.4%)). Both cohorts also had a group with symptoms developing in midlife; however for this group in the NCDS by their 50’s psychological distress was decreasing ‘midlife-onset decreasing’ n=1375, 11.9%) and in the BCS70 the onset of symptoms were severe and increasing ‘midlife-onset decreasing’ n= 961, 9.4%). The final group in the NCDS was repeated minor symptoms ‘stable-low symptoms’ (n=2226, 19.2%), while in the BCS70 the fifth grouping had early adult-onset and more favorable outcomes ‘early adult-onset decreasing’ (BCS70=1114, 10.9%).

These differences may be due to cohort effects, for example the period 1990-1993 was marked by a long recession when the BCS70 cohort members would have been new entrants to the labor market,^8^ perhaps resulting in increased levels of distress for some.

**Table S6: Mean, SE and 95% CIs for mental health outcomes for psychological distress trajectories in the** **NCDS and BCS70**

|  | Psychological distress | | | Life satisfaction | | | Loneliness | | |
| --- | --- | --- | --- | --- | --- | --- | --- | --- | --- |
|  | Wave 1 | Wave 2 | Wave 3 | Wave 1 | Wave 2 | Wave 3 | Wave 1 | Wave 2 | Wave 3 |
| NCDS mental health trajectories  Total  No symptoms  Stable-low symptoms  Adult-onset decreasing  Midlife-onset decreasing  Stable-high symptoms  N: 15,291 | 1.72 (0.06)  [1.60, 1.85]  0.79 (0.06)  [0.67, 0.92]  1.41 (0.06)  [1.28, 1.54]  1.87 (0.09)  [1.68, 2.05]  2.07 (0.09)  [1.89, 2.26]  3.63 (0.09)  [3.45, 3.80] | 2.03 (0.05)  [1.93, 2.12]  1.01 (0.05)  [0.91, 1.11]  1.65 (0.04)  [1.56, 1.74]  2.20 (0.09)  [2.01, 2.39]  2.44 (0.09)  [2.25, 2.62]  4.07 (0.10)  [3.87, 4.27] | 1.76 (0.04)  [1.68, 1.84]  0.80 (0.04)  [0.73, 0.87]  1.46 (0.05)  [1.36, 1.57]  1.97 (0.08)  [1.81, 2.14]  2.16 (0.08)  [2.00, 2.33]  3.62 (0.07)  [3.49, 3.77] | 7.22 (0.06)  [7.00, 7.24]  7.62 (0.06)  [7.51, 7.74]  7.34 (0.09)  [7.16, 7.52]  7.16 (0.08)  [7.00, 7.32]  6.84 (0.11)  [6.61, 7.07]  6.02 (0.09)  [5.84, 6.21] | 6.60 (0.07)  [6.47, 6.74]  7.13 (0.06)  [7.01,7.24]  6.99 (0.07)  [6.86, 7.13]  6.53 (0.13)  [6.28, 6.79]  6.22 (0.12)  [5.98, 6.46]  5.49 (0.14)  [5.19, 5.78] | 6.93 (0.05)  [6.83, 7.03]  7.42 (0.05)  [7.31, 7.53]  6.93 (0.06)  [6.81, 7.05]  6.83 (0.09)  [6.66, 7.00]  6.71 (0.08)  [6.56, 6.87]  6.09 (0.10)  [5.90, 6.29] | 4.41 (0.05)  [4.31, 4.51]  3.89 (0.04)  [3.80, 3.98]  4.22 (0.06)  [4.11, 4.34]  4.44 (0.10)  [4.24, 4.64]  4.73 (0.08)  [4.57, 4.89]  5.43 (0.09)  [5.25, 5.61] | 4.44 (0.04)  [4.36, 4.53]  3.94 (0.04)  [3.87, 4.02]  4.20 (0.05)  [4.09, 4.30]  4.55 (0.09)  [4.37, 4.72]  4.73 (0.07)  [4.58, 4.88]  5.45 (0.09)  [5.27, 5.62] | 4.51 (0.03)  [4.44, 4.58]  3.98 (0.03)  [3.91, 4.04]  4.39 (0.04)  [4.30, 4.48]  4.64 (0.07)  [4.51, 4.78]  4.79 (0.07)  [4.64, 4.93]  5.47 (0.06)  [5.35, 5.58] |
| BCS70 mental health trajectories  Total  No symptoms  Early adult-onset decreasing  Adult- onset decreasing  Midlife-onset increasing  Stable-high symptoms  N: 16,128 | 2.03 (0.06)  [1.90, 2.14]  0.98 (0.05)  [0.88, 1.08]  2.06 (0.09)  [1.89, 2.23]  2.22 (0.10)  [2.02, 2.41]  3.22 (0.10)  [3.01, 3.43]  4.29 (0.13)  [4.03, 4.55] | 2.25 (0.04)  [2.17, 2.33]  1.08 (0.04)  [1.00, 1.16]  2.27 (0.07)  [2.11, 2.43]  2.63 (0.08)  [2.48, 2.79]  3.61 (0.09)  [3.43, 3.80]  4.65 (0.09)  [4.47, 4.83] | 2.09 (0.05)  [2.00, 2.19]  1.02 (0.05)  [0.91, 1.13]  2.15 (0.08)  [1.99, 2.31]  2.21 (0.09)  [2.02, 2.40]  3.46 (0.08)  [3.29, 3.62]  4.38 (0.10)  [4.18, 4.58] | 6.96 (0.07)  [6.81, 7.11]  7.51 (0.07)  [7.37, 7.64]  6.95 (0.11)  [6.73, 7.17]  6.85 (0.13)  [6.58, 7.11]  6.48 (0.10)  [6.28, 6.69]  5.72 (0.15)  [5.41, 6.02] | 6.79 (0.05)  [6.69, 6.89]  7.44 (0.04)  [7.35, 7.53]  6.82 (0.10)  [6.62, 7.02]  6.57 (0.11)  [6.35, 6.79]  6.12 (0.10)  [5.93, 6.31]  5.38 (0.09)  [5.18, 5.57] | 6.70 (0.05)  [6.60, 6.80]  7.22 (0.05)  [7.11, 7.33]  6.68 (0.09)  [6.49, 6.87]  6.64 (0.11)  [6.42, 6.85]  6.22 (0.10)  [6.03, 6.41]  5.52 (0.11)  [5.30, 5.74] | 4.38 (0.05)  [4.29, 4.48]  3.87 (0.05)  [3.78, 3.97]  4.54 (0.08)  [4.38, 4.71]  4.59 (0.08)  [4.41, 4.76]  4.87 (0.08)  [4.71, 5.03]  5.40 (0.11)  [5.17, 5.63] | 4.50 (0.03)  [4.43, 4.57]  3.91 (0.04)  [3.83, 3.99]  4.52 (0.08)  [4.37, 4.67]  4.66 (0.08)  [4.50, 4.81]  5.04 (0.08)  [4.89, 5.20]  5.79 (0.07)  [5.66, 5.93] | 4.55 (0.04)  [4.46, 4.63]  4.01 (0.08)  [3.93, 4.09]  4.66 (0.08)  [4.51, 4.81]  4.71 (0.09)  [4.53, 4.89]  4.98 (0.07)  [4.84, 5.13]  5.70 (0.10)  [5.50, 5.90] |

**Table S7: Influence of psychological distress trajectories on mental health outcomes during the COVID19 pandemic using continuous imputed data (Life satisfaction squared, psychological distress and loneliness treated as count data)**

|  | Psychological distress  Poisson regression coefficient [95% C’s] | | | | | | Life satisfaction  LS^2^ OLS, [95% CIs] | | | | | | Loneliness  Poisson regression coefficient, [95% CIs] | | | | | |
| --- | --- | --- | --- | --- | --- | --- | --- | --- | --- | --- | --- | --- | --- | --- | --- | --- | --- | --- |
|  | Wave 1 | | Wave 2 | | Wave 3 | | Wave 1 | Wave 2 | | | Wave 3 | | Wave 1 | | Wave 2 | | Wave 3 | |
| NCDS mental health trajectories  Ref: No symptoms |  | |  | |  | |  |  | | |  | |  | |  | |  |  |
| Stable-low symptoms | 0.42  [0.34, 0.49] | | 0.38  [0.30, 0.45] | | 0.44  [0.37, 0.51] | | -4.00  [-5.87, -2.13] | -3.41  [-5.23, -1.59] | | | -6.04  [-7.83, -4.26] | | 0.08  [0.05, 0.10] | | 0.06  [0.02, 0.09] | | 0.09  [0.06, 0.12] | |
| Adult-onset decreasing | 0.62  [0.52, 0.72] | | 0.59  [0.50, 0.68] | | 0.68  [0.61, 0.74] | | -5.81  [-7.97, -3.64] | -7.36  [-9.55, -5.18] | | | -7.40  [-9.59, -5.21] | | 0.11  [0.07, 0.16] | | 0.13  [0.09,0.17] | | 0.13  [0.10, 0.17] | |
| Midlife-onset decreasing | 0.75  [0.66, 0.83] | | 0.72  [0.65, 0.80] | | 0.79  [0.73, 0.85] | | -10.16  [-13.02, -7.30] | -11.57  [-14.03, -9.10] | | | -9.03  [-11.08, -6.97] | | 0.19  [0.15, 0.22] | | 0.17  [0.14, 0.21] | | 0.17  [0.14, 0.21] | |
| Stable-high symptoms | 1.21  [1.12, 1.31] | | 1.15  [1.09, 1.21] | | 1.22  [1.16, 1.28] | | -18.82  [-21.20, 16.44] | -18.81  [-20.90, -16.71] | | | -15.88  [-17.84, -13.91] | | 0.31  [0.28, 0.34] | | 0.30  [0.26, 0.33] | | 0.29  [0.26, 0.31] | |
| N 15,291 |  | |  | |  | |  |  | | |  | |  | |  | |  | |
| BCS70 mental health trajectories  Ref: No symptoms |  | |  | |  | |  |  | | |  | |  | |  | |  | |
| Early adult-onset decreasing | 0.57  [0.50, 0.64] | | 0.58  [0.52, 0.65] | | 0.58  [0.49, 0.66] | | -6.91  [-9.47, -4.34] | -8.28  [-10.37, -6.19] | | | -6.48  [-9.07, -3.90] | | 0.15  [0.11, 0.19] | | 0.14  [0.11, 0.18] | | 0.15  [0.10, 0.19] | |
| Adult-onset decreasing | 0.64  [0.57, 0.72] | | 0.73  [0.66, 0.80] | | 0.62  [0.53, 0.71] | | -7.90  [-10.28, -5.51] | -11.73  [-14.31, -9.14] | | | -7.86  [-10.35, -5.37] | | 0.16  [0.12, 0.21] | | 0.18  [0.14, 0.22] | | 0.16  [0.12, 0.20] | |
| Midlife-onset increasing | 0.99  [0.91, 1.08] | | 1.03  [0.96, 1.10] | | 1.03  [0.97, 1.10] | | -13.29  [-16.14, -10.43] | -17.05  [-20.15, -13.96] | | | -12.58  [-15.18, -9.98] | | 0.22  [0.18, 0.26] | | 0.25  [0.20, 0.29] | | 0.21  [0.17, 0.25] | |
| Stable-high symptoms | 1.25  [1.17, 1.32] | | 1.27  [1.22, 1.32] | | 1.25  [1.20, 1.31] | | -20.66  [-23.92, -17.40] | -24.11  [-26.30, -21.92] | | | -20.05  [-22.48, -17.62] | | 0.32  [0.28, 0.36] | | 0.38  [0.35, 0.42] | | 0.35  [0.32, 0.38] | |
| N: 16,128 |  |  | |  | |  | | |  |  | |  | |  | |  | |  |

Psychological distress and Loneliness reported as poisson regression coefficients. Life satisfaction squared and OLS regression, , 95% confidence intervals (95% CIs)

Parameters are adjusted for sex, breastfed, mother smoked during pregnancy, gestation period, birthweight, parental social class at 0, parental education at 0, housing tenure at 7/5, access to house amenities at 7/5, crowding at age 0, 7/5 and 11/10, parents marital status at 0, maternal age at birth, mother worked in first five years, separated from child for more than a month <age 5, read to at 7/5, CM wet the bed at 7/5, had any medical conditions at 7/5, Body Mass Index (BMI) at 11/10, and cognitive ability at 7/5 and 11/10.

Wave 1 (May 2020), Wave 2 (September to October 2020) and Wave 3 (February-March 2021)

**Table S8: Influence of psychological distress trajectories on the predicted probability of mental health outcomes at different time-points during the COVID19 pandemic**

|  | Psychological distress  PP % (SE), [95% CIs] | | | Life satisfaction  RR (SE), [95% CIs] | | | | | | Loneliness  RR (SE), [95% CIs] | | | | | | | |
| --- | --- | --- | --- | --- | --- | --- | --- | --- | --- | --- | --- | --- | --- | --- | --- | --- | --- |
|  | Wave 1 | Wave 2 | Wave 3 | Wave 1 | | Wave 2 | | | Wave 3 | Wave 1 | | Wave 2 | | Wave 3 | | |  |
| NCDS mental health trajectories |  |  |  |  | |  | | |  |  | |  | |  |  |  |  |
| No symptoms | 3.69 (0.01)  [2.77, 4.90] | 5.68 (0.01)  [4.53, 7.14] | 5.80 (0.01)  [4.86, 6.93] | 76.50 (0.02)  [72.87, 80.32] | | 78.61 (0.01)  [76.33, 80.95] | | | 71.87 (0.01)  [69.77, 74.04] | 14.54 (0.01)  [12.07, 17.50] | | 12.92 (0.01)  [11.03, 15.13] | | 17.64 (0.01)  [15.83, 19.65] | | |  |
| Stable-low symptoms | 8.86 (0.01)  [6.70, 11.72] | 10.34 (0.01)  [8.87, 12.06] | 12.42 (0.01)  [10.38, 14.87] | 72.02 (0.02)  [68.54, 75.66] | | 74.47 (0.01)  [71.80, 77.23] | | | 64.83 (0.01)  [61.98, 67.82] | 21.22 (0.01)  [18.43, 24.42] | | 19.34 (0.01)  [17.09, 21.89] | | 27.21 (0.01)  [24.87, 29.77] | | |  |
| Adult-onset decreasing | 15.49 (0.02)  [11.47, 20.91] | 18.54 (0.02)  [15.11, 22.76] | 21.42 (0.02)  [17.44, 26.31] | 65.85 (0.03)  [59.94, 72.35] | | 63.51 (0.02)  [59.38, 67.94] | | | 59.25 (0.02)  [54.66, 64.23] | 24.13 (0.02)  [19.94, 29.21] | | 23.51 (0.02)  [19.52, 28.31] | | 33.10 (0.02)  [29.66, 36.94] | | |  |
| Midlife-onset decreasing | 21.38 (0.03)  [16.74, 27.31] | 22.85 (0.02)  [20.01, 26.09] | 24.30 (0.02)  [20.96, 28.17] | 62.57 (0.03)  [57.60, 67.96] | | 60.98 (0.02)  [56.90, 65.34] | | | 58.45 (0.02)  [54.89, 62.24] | 35.35 (0.03)  [30.37, 41.14] | | 31.01 (0.02)  [27.68, 34.75] | | 37.90 (0.02)  [33.88, 42.38] | | |  |
| Stable-high symptoms | 39.71 (0.04)  [32.55, 48.44] | 47.03 (0.02)  [43.09, 51.34] | 49.73 (0.02)  [45.80, 53.99] | 46.06 (0.03)  [39.49, 53.73] | | 48.05 (0.03)  [43.17, 53.49] | | | 43.71 (0.02)  [40.43, 47.26] | 46.71 (0.03)  [41.02, 53.21] | | 41.83 (0.02)  [37.97, 46.09] | | 50.25 (0.02)  [46.06, 54.81] | | |  |
| N: 15,291 |  |  |  |  | |  | | |  |  | |  | |  | | |  |
| BCS70 mental health trajectories |  |  |  |  | |  | | |  |  | |  | |  | | |  |
|  |  |  |  |  | |  | | |  |  | |  | |  | | |  |
| No symptoms | 5.40 (0.01)  [3.96, 7.36] | 7.00 (0.01)  [5.87, 8.32] | 8.68 (0.01)  [7.04, 10.70] | 76.07 (0.02)  [72.86, 79.43] | | 74.09 (0.01)  [71.47, 76.82] | | | 68.97 (0.01)  [66.38, 71.67] | 15.80 (0.01)  [13.49, 18.51] | | 17.45 (0.01)  [15.28, 10.91] | | 19.24 (0.01)  [17.05, 21.71] | | |  |
| Early adult-onset decreasing | 17.45 (0.02)  [13.57, 22.43] | 22.40 (0.02)  [19.27, 26.04] | 23.45 (0.02)  [20.31, 27.08] | 65.15 (0.02)  [60.93, 69.67] | | 61.98 (0.03)  [56.78, 67.66] | | | 58.56 (0.02)  [54.91, 62.44] | 29.54 (0.02)  [25.74, 33.89] | | 31.25 (0.02)  [27.43, 35.60] | | 33.84 (0.02)  [29.54, 38.78] | | |  |
| Adult-onset decreasing | 22.23 (0.03)  [16.84, 29.34] | 26.73 (0.02)  [22.49, 31.77] | 26.92 (0.03)  [22.19, 32.66] | 62.95 (0.03)  [57.68, 68.71] | | 56.51 (0.03)  [51.64, 61.85] | | | 54.70 (0.03)  [49.37, 60.61] | 29.46 (0.02)  [24.84, 34.92] | | 33.90 (0.03)  [28.34, 40.56] | | 35.93 (0.02)  [31.47, 41.02] | | |  |
| Midlife-onset increasing | 42.71 (0.03)  [36.85, 49.50] | 46.67 (0.02)  [42.34, 51.44] | 47.92 (0.02)  [43.17, 53.20] | 52.10 (0.03)  [46.70, 58.13] | | 46.19 (0.02)  [41.79, 51.06] | | | 46.58 (0.02)  [42.47, 51.09] | 36.79 (0.02)  [32.74, 41.34] | | 41.60 (0.02)  [37.24, 46.48] | | 41.01 (0.02)  [36.70, 45.84] | | |  |
| Stable-high symptoms | 58.45 (0.04)  [50.72, 67.35] | 65.71 (0.02)  [60.96, 70.84] | 64.57 (0.02)  [59.62, 69.93] | 37.98 (0.02)  [33.32, 43.28] | | 29.48 (0.02)  [25.32, 34.32] | | | 34.58 (0.02)  [30.36, 39.37] | 48.36 (0.03)  [42.25, 55.36] | | 59.17 (0.03)  [54.09, 64.71] | | 58.23 (0.03)  [52.54, 64.52] | | |  |
| N: 16,128 |  |  |  | |  | |  |  | | |  | |  | |  |  |  |

Reported as predicted probabilities, standard error (SE), 95% confidence intervals (95% CIs)

Outcomes are psychological distress ≥4 or not, life satisfaction ≥7 or not. loneliness ≥6 or not

Parameters are adjusted for sex, breastfed, mother smoked during pregnancy, gestation period, birthweight, parental social class at 0, parental education at 0, housing tenure at 7/5, access to house amenities at 7/5, crowding at age 0, 7/5 and 11/10, parents marital status at 0, maternal age at birth, mother worked in first five years, separated from child for more than a month <age 5, read to at 7/5, CM wet the bed at 7/5, had any medical conditions at 7/5, Body Mass Index (BMI) at 11/10, and cognitive ability at 7/5 and 11/10.

Wave 1 (May 2020), Wave 2 (September to October 2020) and Wave 3 (February-March 2021)

**Table S9.1: Influence of psychological distress trajectories on the relative risk (RR) of mental health outcomes during the COVID19 pandemic in the NCDS, controlling for mid-life factors**

|  | Psychological distress  RR (SE), [95% CIs] | | | | Life satisfaction  RR (SE), [95% CIs] | | | | Loneliness  RR (SE), [95% CIs] | | | |
| --- | --- | --- | --- | --- | --- | --- | --- | --- | --- | --- | --- | --- |
| Wave | Early Life | +Mid-life mediators | +Mental Health | +Mid-life Mediators & Mental Health | Early Life | +Mid-life mediators | +Mental health | +Mid-life Mediators & Mental Health | Early Life | +Mid-life mediators | +Mental Health | +Mid-life Mediators & Mental Health |
| Wave 1: NCDS mental health trajectories  Ref: No symptoms |  |  |  |  |  |  |  |  |  |  |  |  |
| Stable-low symptoms | 2.40 (0.32)  [1.85, 3.13] | 2.23 (0.37)  [1.60, 3.10] | 1.91 (0.31)  [1.40,2.62] | 1.89 (0.30)  [1.37, 2,61] | 0.94 (0.02)  [0.90, 0.99] | 0.97 (0.02)  [0.93, 1.01] | 1.00 (0.02)  [0.95,1.04] | 1.00 (0.02)  [0.96, 1.05] | 1.46 (0.14)  [1.21, 1.76] | 1.35 (0.13)  [1.12, 1.63] | 1.26 (0.12)  [1.05,1.52] | 1.24 (0.12)  [1.03, 1.50] |
| Adult-onset decreasing | 4.20 (0.70)  [3.04, 5.82] | 3.42 (0.57)  [2.44, 4.79] | 2.79 (0.45)  [2.03,3.82] | 2.60 (0.42)  [1.88, 3.60] | 0.86 (0.03)  [0.80, 0.92] | 0.92 (0.04)  [0.84, 1.00] | 0.94 (0.04)  [0.86,1.02] | 0.97 (0.04)  [0.89, 1.06] | 1.66 (0.19)  [1.33, 2.08] | 1.54 (0.15)  [1.26, 1.88] | 1.39 (0.15)  [1.13,1.71] | 1.33 (0.14)  [1.09, 1.64] |
| Midlife-onset decreasing | 5.80 (0.76)  [4.49, 7.49] | 4.99 (0.82)  [3.57, 6.97] | 3.59 (0.60)  [2.58,4.99] | 3.44 (0.58)  [2.45, 4.82] | 0.82 (0.03)  [0.76, 0.88] | 0.89 (0.03)  [0.84, 0.95] | 0.93 (0.03)  [0.88,0.99] | 0.96 (0.03)  [0.90, 1.03] | 2.43 (0.20)  [2.07, 2.85] | 2.13 (0.17)  [1.81, 2.50] | 1.84 (0.15)  [1.58,2.16] | 1.75 (0.14)  [1.49, 2.05] |
| Stable-high symptoms | 10.78 (1.34)  [8.45, 13.74] | 8.25 (1.20)  [6.16, 11.06] | 4.20 (0.63)  [3.13,5.64] | 3.93 (0.58)  [2.92, 5.30] | 0.60 (0.03)  [0.54, 0.68] | 0.76 (0.03)  [0.69, 0.82] | 0.84 (0.04)  [0.77,0.92] | 0.88 (0.04)  [0.81, 0.96] | 3.21 (0.22)  [2.80, 3.68] | 2.38 (0.23)  [1.96, 2.90] | 1.78 (0.19)  [1.44,2.21] | 1.60 (0.18)  [1.28, 2.00] |
|  |  |  |  |  |  |  |  |  |  |  |  |  |
| Wave 2: NCDS mental health trajectories  Ref: No symptoms |  |  |  |  |  |  |  |  |  |  |  |  |
| Stable-low symptoms | 1.82 (0.23)  [1.43, 2.32] | 1.64 (0.20)  [1.29, 2.08] | 1.44 (0.17)  [1.14,1.83] | 1.40 (0.17)  [1.10, 1.78] | 0.95 (0.02)  [0.91, 0.98] | 0.97 (0.02)  [0.93, 1.02] | 1.01 (0.02)  [0.97,1.06] | 1.01 (0.02)  [0.97, 1.06] | 1.50 (0.12)  [1.28, 1.75] | 1.36 (0.11)  [1.16, 1.59] | 1.27 (0.10)  [1.09,1.48] | 1.24 (0.10)  [1.06, 1.44] |
| Adult-onset decreasing | 3.26 (0.41)  [2.54, 4.18] | 2.80 (0.32)  [2.24, 3.51] | 2.34 (0.27)  [1.88,2.93] | 2.17 (0.25)  [1.73, 2.73] | 0.81 (0.03)  [0.76, 0.86] | 0.87 (0.03)  [0.81, 0.93] | 0.90 (0.03)  [0.84,0.97] | 0.92 (0.03)  [0.86, 0.99] | 1.82 (0.20)  [1.47, 2.25] | 1.58 (0.14)  [1.32, 1.89] | 1.42 (0.14)  [1.17,1.72] | 1.36 (0.12)  [1.13, 1.63] |
| Midlife-onset decreasing | 4.02 (0.48)  [3.18, 5.09] | 3.32 (0.33)  [2.72, 4.04] | 2.53 (0.26)  [2.06,3.10] | 2.36 (0.24)  [1.92, 2.90] | 0.78 (0.03)  [0.73, 0.83] | 0.84 (0.03)  [0.79, 0.89] | 0.89 (0.03)  [0.84,0.95] | 0.92 (0.03)  [0.86, 0.98] | 2.40 (0.19)  [2.05, 2.81] | 2.05 (0.16)  [1.75, 2.40] | 1.78 (0.15)  [1.51,2.09] | 1.66 (0.14)  [1.41, 1.95] |
| Stable-high symptoms | 8.28 (0.93)  [6.64, 10.32] | 5.96 (0.58)  [4.90, 7.24] | 3.30 (0.35)  [2.69,4.05] | 3.01 (0.32)  [2.44, 3.72] | 0.61 (0.03)  [0.56, 0.67] | 0.73 (0.03)  [0.67, 0.81] | 0.84 (0.04)  [0.77,0.92] | 0.89 (0.04)  [0.81, 0.97] | 3.24 (0.29)  [2.71, 3.86] | 2.37 (0.18)  [2.03, 2.75] | 1.73 (0.15)  [1.45,2.06] | 1.54 (0.14)  [1.29, 1.84] |
|  |  |  |  |  |  |  |  |  |  |  |  |  |
| Wave 3: NCDS mental health trajectories  Ref: No symptoms |  |  |  |  |  |  |  |  |  |  |  |  |
| Stable-low symptoms | 2.14 (0.22)  [1.75, 2,61] | 2.00 (0.23)  [1.59, 2.50] | 1.79 (0.20)  [1.44,2.22] | 1.73 (0.20)  [1.39, 2,17] | 0.90 (0.02)  [0.86, 0.94] | 0.93 (0.02)  [0.89, 0.98] | 0.97 (0.02)  [0.92,1.01] | 0.98 (0.02)  [0.93, 1.02] | 1.54 (0.08)  [1.38, 1.72] | 1.45 (0.08)  [1.29, 1.63] | 1.40 (0.08)  [1.24,1.57] | 1.36 (0.08)  [1.21, 1.52] |
| Adult-onset decreasing | 3.69 (0.37)  [3.03, 4.49] | 3.13 (0.34)  [2.51, 3.90] | 2.65 (0.29)  [2.14,3.29] | 2.48 (0.28)  [1.99, 3.10] | 0.82 (0.03)  [0.76, 0.89] | 0.88 (0.03)  [0.82, 0.94] | 0.92 (0.03)  [0.86,0.99] | 0.94 (0.03)  [0.88, 1.02] | 1.88 (0.11)  [1.67, 2.10] | 1.68 (0.11)  [1.48, 1.92] | 1.59 (0.10)  [1.41,1.79] | 1.51 (0.10)  [1.33, 1.71] |
| Midlife-onset decreasing | 4.19 (0.40)  [3.46, 5.07] | 3.50 (0.42)  [2.75, 4.46] | 2.73 (0.33)  [2.16,3.46] | 2.57 (0.32)  [2.01, 3.30] | 0.81 (0.03)  [0.76, 0.87] | 0.88 (0.03)  [0.83, 0.94] | 0.94 (0.03)  [0.89,1.00] | 0.98 (0.03)  [0.91, 1.04] | 2.15 (0.15)  [1.87, 2.47] | 1.86 (0.12)  [1.63, 2.13] | 1.72 (0.11)  [1.52,1.95] | 1.61 (0.10)  [1.39, 1.85] |
| Stable-high symptoms | 8.57 (0.71)  [7.27, 10.10] | 6.50 (0.69)  [5.24, 8.06] | 3.82 (0.41)  [3.09,4.72] | 3.53 (0.40)  [2.81, 4.43] | 0.61 (0.02)  [0.56, 0.66] | 0.71 (0.03)  [0.65, 0.78] | 0.83 (0.04)  [0.75,0.91] | 0.88 (0.04)  [0.80, 0.97] | 2.85 (0.15)  [2.56, 3,17] | 2.17 (0.14)  [1.92, 2.46] | 1.80 (0.11)  [1.59,2.04] | 1.60 (0.11)  [1.39, 1.84] |
|  |  |  |  |  |  |  |  |  |  |  |  |  |

Reported as Relative Risk (RR), standard error (SE), 95% confidence intervals (95% CIs). N: 15,291

Outcomes are psychological distress ≥4 or not, life satisfaction ≥7 or not. loneliness ≥6 or not

Parameters are adjusted for sex, breastfed, mother smoked during pregnancy, gestation period, birthweight, parental social class at 0, parental education at 0, housing tenure at 7, access to house amenities at 7, crowding at age 0, 7 and 11, parents marital status at 0, maternal age at birth, mother worked in first five years, separated from child for more than a month <age 5, read to at 7, CM wet the bed at 7, had any medical conditions at 7, Body Mass Index (BMI) at 11, and cognitive ability at 7 and 11. Midlife mediators adjusted for social class, highest educational attainment, income, employment status, housing tenure, cognitive function, general health, smoked, drinking, BMI, household size, number of children and partnership status. Midlife mental health: Psychological distress at age 50/55 in the NCDS. Mid-life Mediators & Mental Health: Adjusted on all variables.

Wave 1 (May 2020), Wave 2 (September to October 2020) and Wave 3 (February-March 2021)

**Table S9.2: Influence of psychological distress trajectories on the relative risk (RR) of mental health outcomes during the COVID19 pandemic in the BCS70, controlling for mid-life factors**

|  | Psychological distress  RR (SE), [95% CIs] | | | | | | | Life satisfaction  RR (SE), [95% CIs] | | | | | | | Loneliness  RR (SE), [95% CIs] | | | | | | |
| --- | --- | --- | --- | --- | --- | --- | --- | --- | --- | --- | --- | --- | --- | --- | --- | --- | --- | --- | --- | --- | --- |
|  | Early Life | | +Mid-life mediators | | +Mental Health | | +Mid-life Mediators & Mental Health | Early Life | +Mid-life mediators | | | +Mental Health | | +Mid-life mediators & Mental Health | Early Life | +Mid-life mediators | | +Mental Health | | +Mid-life Mediators & Mental Health | |
| Wave 1: BCS70 mental health trajectories  Ref: No symptoms |  | |  | |  | |  |  |  | | |  | |  |  |  | |  | |  | |
| Early adult-onset decreasing | 3.28 (0.46)  [2.48, 4.34] | | 2.71 (0.30)  [2.17, 3.38] | | 2.28 (0.24)  [1.85,2.81] | | 2.18 (0.23)  [1.78, 2.69] | 0.85 (0.04)  [0.77, 0.94] | 0.90 (0.03)  [0.83, 0.97] | | | 0.95 (0.04)  [0.88,1.03] | | 0.96 (0.04)  [0.88, 1.04] | 1.94 (0.19)  [1.60, 2.36] | 1.76 (0.16)  [1.46, 2.12] | | 1.62 (0.15)  [1.35,1.93] | | 1.56 (0.14)  [1.30, 1.87] | |
| Adult-onset decreasing | 3.90 (0.62)  [2.82, 5.39] | | 3.58 (0.53)  [2.64, 4.85] | | 2.98 (0.43)  [2.24,3.96] | | 2.82 (0.42)  [2.09, 3.80] | 0.82 (0.04)  [0.75, 0.90] | 0.86 (0.04)  [0.77, 0.95] | | | 0.90 (0.05)  [0.81,1.01] | | 0.92 (0.05)  [0.82, 1.04] | 2.00 (0.19)  [1.65, 2.42] | 1.73 (0.20)  [1.37, 2.19] | | 1.61 (0.18)  [1.29,2.02] | | 1.51 (0.17)  [1.20, 1.91] | |
| Midlife-onset increasing | 7.67 (0.86)  [6.11, 9.63] | | 6.07 (0.74)  [4.73, 7.78] | | 2.70 (0.33)  [2.12,3.44] | | 2.56 (0.31)  [2.00, 3.26] | 0.67 (0.03)  [0.61, 0.74] | 0.75 (0.04)  [0.69, 0.83] | | | 1.02 (0.06)  [0.91,1.15] | | 0.99 (0.06)  [0.88, 1.11] | 2.38 (0.22)  [1.97, 2.88] | 1.99 (0.17)  [1.67, 2.37] | | 1.20 (0.13)  [0.98,1.48] | | 1.21 (0.12)  [1.00, 1.46] | |
| Stable-high symptoms | 10.79 (1.14)  [8.71,13.36] | | 7.78 (0.85)  [6.24, 9.70] | | 3.13 (0.38)  [2.47,3.97] | | 2.94 (0.36)  [2.29, 3.78] | 0.49 (0.04)  [0.42, 0.57] | 0.63 (0.04)  [0.56, 0.72] | | | 0.82 (0.07)  [0.70,0.97] | | 0.87 (0.07)  [0.74, 1.01] | 3.09 (0.26)  [2.61, 3.67] | 2.20 (0.20)  [1.81, 2.66] | | 1.36 (0.16)  [1.07,1.72] | | 1.25 (0.14)  [0.99, 1.57] | |
|  |  | |  | |  | |  |  |  | | |  | |  |  |  | |  | |  | |
| Wave 2: BCS70 mental health trajectories  Ref: No symptoms |  | |  | |  | |  |  |  | | |  | |  |  |  | |  | |  | |
| Early adult-onset decreasing | 3.08 (0.39)  [2.38, 3.97] | | 2.95 (0.30)  [2.41, 3.61] | | 2.55 (0.26)  [2.10,3.11] | | 2.48 (0.26)  [2.02, 3.05] | 0.84 (0.02)  [0.80, 0.89] | 0.87 (0.03)  [0.82, 0.93] | | | 0.94 (0.03)  [0.87,1.01] | | 0.94 (0.03)  [0.88, 1.01] | 1.77 (0.15)  [1.48, 2.10] | 1.71 (0.15)  [1.42, 2.05] | | 1.56 (0.14)  [1.30,1.87] | | 1.52 (0.14)  [1.26, 1.84] | |
| Adult-onset decreasing | 3.73 (0.37)  [3.07, 4.54] | | 3.33 (0.32)  [2.75, 4.04] | | 2.86 (0.27)  [2.38,3.43] | | 2.77 (0.26)  [2.30, 3.34] | 0.77 (0.03)  [0.70, 0.84] | 0.84 (0.03)  [0.77, 0.92] | | | 0.88 (0.04)  [0.81,0.96] | | 0.91 (0.04)  [0.84, 0.99] | 1.94 (0.16)  [1.65, 2.29] | 1.74 (0.13)  [1.50, 2.02] | | 1.62 (0.12)  [1.39,1.88] | | 1.54 (0.12)  [1.31, 1.81] | |
| Midlife-onset increasing | 6.66 (0.63)  [5.49, 8.01] | | 5.69 (0.51)  [4.76, 6.80] | | 2.96 (0.29)  [2.45,3.59] | | 2.83 (0.28)  [2.32, 3.45] | 0.63 (0.03)  [0.57, 0.70] | 0.70 (0.03)  [0.64, 0.77] | | | 0.98 (0.05)  [0.88,1.09] | | 0.96 (0.05)  [0.87, 1.07] | 2.40 (0.18)  [2.06, 2.79] | 2.07 (0.13)  [1.84, 2.34] | | 1.33 (0.10)  [1.15,1.55] | | 1.30 (0.10)  [1.12, 1.52] | |
| Stable-high symptoms | 9.31 (0.85)  [7.74, 11.21] | | 7.54 (0.58)  [6.46, 8.79] | | 3.55 (0.31)  [3.00,4.20] | | 3.42 (0.30)  [2.87, 4.08] | 0.41 (0.03)  [0.36, 0.47] | 0.53 (0.03)  [0.47, 0.60] | | | 0.71 (0.05)  [0.62,0.83] | | 0.76 (0.05)  [0.66, 0.87] | 3.43 (0.21)  [3.03, 3.89] | 2.70 (0.18)  [2.36, 3.09] | | 1.70 (0.14)  [1.44,2.01] | | 1.59 (0.14)  [1.32, 1.91] | |
|  |  | |  | |  | |  |  |  | | |  | |  |  |  | |  | |  | |
| Wave 3: BCS70 mental health trajectories  Ref: No symptoms |  | |  | |  | |  |  |  | | |  | |  |  |  | |  | |  | |
| Early adult-onset decreasing | 2.67 (0.28)  [2.17, 3.29] | | 2.39 (0.22)  [1.99, 2.86] | | 2.00 (0.19)  [1.55,2.39] | | 1.94 (0.18)  [1.62, 2.34] | 0.85 (0.03)  [0.78, 0.91] | 0.89 (0.03)  [0.83, 0.95] | | | 0.93 (0.03)  [0.86,1.00] | | 0.94 (0.03)  [0.87, 1.01] | 1.78 (0.13)  [1.54, 2.06] | 1.66 (0.13)  [1.42, 1.94] | | 1.53 (0.12)  [1.31,1.79] | | 1.47 (0.12)  [1.26, 1.73] | |
| Adult-onset decreasing | 3.15 (0.32)  [2.57, 3.86] | | 2.78 (0.26)  [2.29, 3.36] | | 2.33 (0.22)  [1.93,2.80] | | 2.23 (0.21)  [1.83, 2.70] | 0.79 (0.03)  [0.73, 0.87] | 0.85 (0.03)  [0.79, 0.92] | | | 0.88 (0.04)  [0.81,0.96] | | 0.91 (0.04)  [0.84, 0.99] | 1.89 (0.15)  [1.62, 2.21] | 1.71 (0.14)  [1.45, 2.02] | | 1.58 (0.14)  [1.34,1.87] | | 1.50 (0.13)  [1.26, 1.78] | |
| Midlife-onset increasing | 5.57 (0.49)  [4.67, 6.65] | | 4.79 (0.33)  [4.18, 5.49] | | 2.18 (0.19)  [184,2.58] | | 2.14 (0.19)  [1.79, 2.55] | 0.69 (0.03)  [0.63, 0.76] | 0.74 (0.03)  [0.68, 0.82] | | | 0.97 (0.06)  [0.87,1.10] | | 0.95 (0.05)  [0.85, 1.07] | 2.14 (0.14)  [1.87, 2.44] | 1.88 (0.14)  [1.63, 2.18] | | 1.18 (0.11)  [0.99,1.41] | | 1.17 (0.11)  [0.97, 1.41] | |
| Stable-high symptoms | 7.56 (0.59)  [6.46, 8.84] | | 5.83 (0.41)  [5.06, 6.71] | | 2.44 (0.22)  [2.05,2.90] | | 2.32 (0.21)  [1.94, 2.77] | 0.49 (0.04)  [0.42, 0.58] | 0.61 (0.03)  [0.55, 0.68] | | | 0.77 (0.05)  [0.67,0.88] | | 0.81 (0.05)  [0.71, 0.93] | 3.07 (0.17)  [2.75, 3.42] | 2.38 (0.15)  [2.09, 2.71] | | 1.49 (0.14)  [1.24,1.78] | | 1.37 (0.12)  [1.14, 1.64] | |
|  |  |  | |  | |  | |  | |  |  | |  | |  | |  | |  | |  |

Reported as Relative Risk (RR), standard error (SE), 95% confidence intervals (95% CIs). N: 16,128

Outcomes are psychological distress ≥4 or not, life satisfaction ≥7 or not. loneliness ≥6 or not

Parameters are adjusted for sex, breastfed, mother smoked during pregnancy, gestation period, birthweight, parental social class at 0, parental education at 0, housing tenure at 5, access to house amenities at 5, crowding at age 0, 5 and 10, parents marital status at 0, maternal age at birth, mother worked in first five years, separated from child for more than a month <age 5, read to at 5, CM wet the bed at 5, had any medical conditions at 5, Body Mass Index (BMI) at 10, and cognitive ability at 5 and 10. Midlife mediators adjusted for social class, highest educational attainment, income, employment status, housing tenure, cognitive function, general health, smoked, drinking, BMI, household size, number of children and partnership status. Midlife mental health: Psychological distress at age 46 in BCS70. Mid-life Mediators & Mental Health: Adjusted on all variables.

Wave 1 (May 2020), Wave 2 (September to October 2020) and Wave 3 (February-March 2021)

**Table S10: Sensitivity analysis for unmeasured confounding E-values**

To investigate if an unmeasured confounder is to explain away the observed effect estimate or reduce it to a particular level we employed the E-Value approach^9,10^ which with minimal assumptions allows researchers to calculate the strength of confounding that is needed to fully explain away their results.

|  | Psychological distress  E-value [95% CI] | | | | | Life satisfaction  E-value [95% CI] | | | | | Loneliness  E-value [95% CIs] | | | | | |
| --- | --- | --- | --- | --- | --- | --- | --- | --- | --- | --- | --- | --- | --- | --- | --- | --- |
|  | Wave 1 | | Wave 2 | | Wave 3 | Wave 1 | | Wave 2 | | Wave 3 | Wave 1 | | Wave 2 | | Wave 3 | |
| NCDS mental health trajectories  Ref: No symptoms |  | |  | |  |  | |  | |  |  | |  | |  |  |
| Stable-low symptoms | 3.19  [2.08, 4.66] | | 2.15  [1.43, 2.96] | | 2.85  [2.13, 3.76] | - | | - | | - | 1.79  [1.21, 2.37] | | 1.79  [1.31, 2.24] | | 2.06  [1.71, 2.41] | |
| Adult-onset decreasing | 4.64  [3.17, 6.66] | | 3.76  [2.85, 4.90] | | 4.40  3.39, 5.65] | - | | 1.37  [1.11, 1.54] | | - | 1.99  [1.40, 2.66] | | 2.24  [1.51, 2.64] | | 2.39  [1.99, 2.81] | |
| Midlife-onset decreasing | 6.34  [4.33, 9.11] | | 4.15  [3.25, 5.25] | | 4.58  [3.43, 6.05] | - | | 1.37  [1.16, 1.54] | | - | 2.90  [2.34, 3.52] | | 2.71  [2.17, 3.31] | | 2.60  [2.13, 3.10] | |
| Stable-high symptoms | 7.32  [5.29, 10.07] | | 5.47  [4.31, 6.90] | | 6.52  [5.07, 8.33] | 1.49  [1.24, 1.67] | | 1.46  [1.21, 1.67] | | 1.49  [1.21,1.69] | 2.58  [1.88, 3.41] | | 2.45  [1.90, 3.08] | | 2.58  [2.13, 3.08] | |
| N 15,291 |  | |  | |  |  | |  | |  |  | |  | |  | |
| BCS70 mental health trajectories  Ref: No symptoms |  | |  | |  |  | |  | |  |  | |  | |  | |
| Early adult-onset decreasing | 3.78  [2.96, 4.82] | | 4.40  [3.46, 5.57] | | 3.29  [2.62, 4.11] | - | | - | | - | 2.49  [1.92, 3.15] | | 2.41  [1.81, 3.08] | | 2.30  [1.83, 2.85] | |
| Adult-onset decreasing | 5.09  [3.60, 7.06] | | 4.98  [4.03, 6.14] | | 3.89  [3.06, 4.84] | - | | 1.40  [1.11, 1.59] | | 1.40  [1.11, 1.59] | 2.39  [1.69, 3.23] | | 2.45  [1.95, 3.02] | | 2.37  [1.83, 2.96] | |
| Midlife-onset increasing | 4.56  [3.41, 5.97] | | 5.11  [4.07, 6.36] | | 3.70  [2.98, 4.54] | - | | - | | - | - | | 1.92  [1.49, 2.41] | | - | |
| Stable-high symptoms | 5.33  [4.01, 7.02] | | 6.30  [5.19, 7.62] | | 4.07  [3.29, 4.98] | - | | 1.79  [1.51, 2.01] | | 1.67  [1.34, 1.90] | - | | 2.56  [1.97, 3.23] | | 2.08  [1.54, 2.66] | |
| N: 16,128 |  |  | |  | |  |  | |  | |  |  | |  | |  |

Reported as E-Values, 95% confidence intervals (95% CIs).’ –' represents outcomes explained by adjusted parameters in model.

Outcomes are psychological distress ≥4 or not, life satisfaction ≥7 or not. loneliness ≥6 or not

Parameters are adjusted for sex, breastfed, mother smoked during pregnancy, gestation period, birthweight, parental social class at 0, parental education at 0, housing tenure at 7/5, access to house amenities at 7/5, crowding at age 0, 7/5 and 11/10, parents marital status at 0, maternal age at birth, mother worked in first five years, separated from child for more than a month <age 5, read to at 7/5, CM wet the bed at 7/5, had any medical conditions at 7/5, Body Mass Index (BMI) at 11/10, and cognitive ability at 7/5 and 11/10. Mid-life social class, highest educational attainment, income, employment status, housing tenure, cognitive function, general health, smoked, drinking, BMI, household size, number of children and partnership status, Midlife psychological distress at age 50/46 in the NCDS and BCS70 respectively.

Wave 1 (May 2020), Wave 2 (September to October 2020) and Wave 3 (February-March 2021)

In Figure S1 we present the point estimates of the association between psychological distress trajectories and mental health at wave 1 during the COVID-19 pandemic in the NCDS. We see that all the midlife mediators point estimates (risk ratios) of confounders in the model range from 0.66 (very good general health, compared to poor) to 1.54 (never worked), whereas the E-Value indicates that a stronger confounder with risk ratio with a magnitude of 7.32 and lower limit of the 95%CI of 5.29 would be needed in order to completely explain away our findings in the ‘stable-high symptoms’ group, compared to the ‘no-symptoms’ group. A risk ratio of 6.34 with a lower limit of the 95% CI of 4.33 in the ‘midlife-onset’, 4.64 with a lower limit of the 95% CI of 3.17 in the ‘adult-onset’ and 3.19 with a lower limit of the 95% CI of 2.08 in the ‘stable-low’ group would be needed to explain away findings.

Figure S1. Point estimates (risk ratios) of midlife confounders and the E – Value of the association between psychological distress trajectories and mental health in May 2020 (wave 1) during the COVID-19 pandemic in NCDS

E-values for each psychological trajectory, compared to no-symptoms

E-values for psychological distress trajectories. Point estimates as ordered in the graph: Midlife mental health. Midlife social class, highest educational attainment, income, employment status, housing tenure, household size, number of children and partnership status, cognitive function, general health, smoked, drinking, BMI. Parameters are adjusted for sex, breastfed, mother smoked during pregnancy, gestation period, birthweight, parental social class at 0, parental education at 0, housing tenure at 7, access to house amenities at 7, crowding at age 0, 7 and 11, parents marital status at 0, maternal age at birth, mother worked in first five years, separated from child for more than a month <age 5, read to at 7, CM wet the bed at 7, had any medical conditions at 7, Body Mass Index (BMI) at 11, and cognitive ability at 7 and 11.

In Figure S2 we present the point estimates in the BCS70 of the association between psychological distress trajectories and mental health at wave 1 during the COVID-19 pandemic. We see that all the midlife mediators point estimates (risk ratios) of confounders in the model range from 0.67 (Excellent general health, compared to poor) to 1.28 (unit increase in mental health at age 46), whereas the E-Value indicates that a stronger confounder with risk ratio with a magnitude of 5.33 and lower limit of the 95%CI of 4.01 would be needed in order to completely explain away our findings in the ‘stable-high symptoms’ group, compared to the ‘no-symptoms’ group. A risk ratio of 4.56 with a lower limit of the 95% CI of 3.41 in the ‘midlife-onset’, 5.09 with a lower limit of the 95% CI of 3.60 in the ‘adult-onset’ and 3.78 with a lower limit of the 95% CI of 2.96 in the ‘early adult-onset’ group would be needed to explain away findings.

Figure S2. Point estimates (risk ratios) of midlife confounders and the E – Value of the association between psychological distress trajectories and mental health in May 2020 (wave 1) during the COVID-19 pandemic in BCS70

E-values for each psychological trajectory, compared to no-symptoms

E-values for psychological distress trajectories. Point estimates as ordered in the graph: Midlife mental health. Midlife social class, highest educational attainment, income, housing tenure, household size, number of children and partnership status cognitive function, general health, smoked, drinking, BMI, Parameters are adjusted for sex, breastfed, mother smoked during pregnancy, gestation period, birthweight, parental social class at 0, parental education at 0, housing tenure at 5, access to house amenities at 5, crowding at age 0, 5 and 10, parents marital status at 0, maternal age at birth, mother worked in first five years, separated from child for more than a month <age 5, read to at 5, CM wet the bed at 5, had any medical conditions at 5, Body Mass Index (BMI) at 10, and cognitive ability at 5 and 10.

**Table S11.1 and 11.2: comparison of different psychological distress trajectories**

Main findings from Influence of psychological distress trajectories on the RR of mental health outcomes during the COVID19 pandemic, comparing different psychological distress trajectories.

Comparing all the psychological distress trajectories with each other, the relative risk of the association of mental health outcomes at each stage in the pandemic were significantly different at the 95% level for most comparisons (see Table 6a (NCDS) and Table 6b (BCS70). The exceptions, were the comparable associations of high psychological distress (RR: 1.23, p=.09 in wave 2; RR:1.14, p=.22), life satisfaction (RR: 0.95, p=.25 in wave 1; RR: 0.96, p=.38 in wave 2; RR:0.98, p=.75 in wave 3) and feelings of loneliness (RR:1.14, p=.06 in wave 3) with ‘adult-onset’ compared to ‘midlife-onset’ groups; and feelings of loneliness associated with the ‘stable-low symptoms’ and ‘adult-onset’ groups (RR: 1.14, p=.23 in wave 1; RR:1.22, p=.06 in wave 2) during the pandemic in the NCDS.

Similarities were also found in the BCS70 for the associated risk of high psychological distress (RR: 1.27 p=.16 in wave 1; RR:1.06, p=.61 in wave 2; RR:1.15 , p=.19 in wave 3), lower life satisfaction (RR: .97 p=.49 in wave 1; RR:.91, p=.18 in wave 2; RR: .93, p=.25 in wave 3) and loneliness (RR: 1.00, p=.98 in wave 1; RR:1.06 p=.61 in wave 2; RR: 1.06, p=.48 in wave 3) with the ‘early adult-onset’ and ‘adult-onset’. By wave 3 the probability of feelings of loneliness were similar for the adult-onset and midlife-onset groups in the BCS70 (RR: 1.14, p=.09).

**Table S11.1: Influence of psychological distress trajectories on the RR of mental health outcomes during the COVID19 pandemic, comparing different psychological distress trajectories in the NCDS**

|  | Wave 1  RR, [95% CI] | | | | Wave 2  RR, [95% CI] | | | | Wave 3  RR, [95% CI] | | | |  |
| --- | --- | --- | --- | --- | --- | --- | --- | --- | --- | --- | --- | --- | --- |
|  | NS | SLS | AOD | MOD | NS | SLS | AOD | MOD | NS | SLS | AOD | MOD | |
| Psychological distress |  |  |  |  |  |  |  |  |  |  |  |  | |
| SLS | 2.40  [1.85, 3.13] | - |  |  | 1.82  [1.43, 2,32] | - |  |  | 2.14  [1.76, 2.60] | - |  |  | |
| AOD | 4.20  [3.04, 5.82] | 1.75  [1.30, 2.36] | - |  | 3.26  [2.54, 4.18] | .1.79  [1.42, 2.27] | - |  | 3.69  [3.04, 4.48] | 1.72  [1.38, 2.16] | - |  | |
| MOD | 5.80  [4.49, 7.49] | 2.41  [1.96, 2.97] | 1.38  [1.08, 1.77] | - | 4.02  [3.18, 5.09] | 2.21  [1.82, 2.68] | .1.23  [0.97, 1.56] | - | 4.19  [3.47, 5.05] | 1.96  [1.56, 2.45] | 1.14  [0.93, 1.39] | - | |
| SHS | 10.78  [8.45, 13.74] | 4.48  [3.46, 5.81] | 2.56  [2.06, 3.20] | 1.86  [1.50, 2.30] | 8.28  [6.64, 10.32] | 4.55  [3.83, 5.40] | 2.54  [2.09, 3.08] | 2.06  [1.77, 2.39] | 8.57  [7.29, 10.08] | 4.01  [3.39, 4.73] | 2.32  [1.96, 2.75] | 2.05  [1.78, 2.35] | |
| Life satisfaction |  |  |  |  |  |  |  |  |  |  |  |  | |
| SLS | 0.94  [0.90, 0.99] | - |  |  | 0.95  [0.91, 0.98] | - |  |  | 0.90  [0.86, 0.94] | - |  |  | |
| AOD | 0.86  [0.80, 0.92] | 0.91  [0.84, 0.99] | - |  | 0.81  [0.76, 0.86] | .0.85  [0.79, 0.92] | - |  | 0.83  [0.77, 0.89] | 0.91  [0.85, 0.99] | - |  | |
| MOD | 0.82  [0.76, 0.88] | 0.87  [0.81, 0.94] | 0.95  [0.87, 1.04] | - | 0.78  [0.73, 0.83] | 0.82  [0.76, 0.88] | 0.96  [0.88, 1.05] | - | 0.81  [0.76, 0.87] | 0.90  [0.83, 0.97] | 0.98  [0.88, 1.09] | - | |
| SHS | 0.60  [0.54, 0.68] | 0.64  [0.56, 0.73] | 0.70  [0.62, 0.79] | 0.74  [0.65, 0.84] | 0.61  [0.56, 0.67] | 0.65  [0.58, 0.72] | 0.76  [0.68, 0.84] | 0.79  0.71, 0.87] | 0.61  [0.56, 0.65] | 0.67  [0.62, 0.73] | 0.73  [0.68, 0.80] | 0.75  [0.68, 0.83] | |
| Loneliness |  |  |  |  |  |  |  |  |  |  |  |  | |
| SLS | 1.46  [1.21, 1.76] | - |  |  | 1.50  [1.28, 1.75] | - |  |  | 1.54  [1.38, 1.72] | - |  |  | |
| AOD | 1.66  [1.33, 2.08] | 1.14  [0.92, 1.40] | - |  | 1.82  [1.47, 2.25] | 1.22  [1.00, 1.48] |  |  | 1.88  [1.68, 2.10] | 1.22  [1.08, 1.37] | - |  | |
| MOD | 2.43  [2.07, 2.85] | 1.67  [1.43, 1.94] | 1.46  [1.18, 1.82] | - | 2.40  [2.05, 2.81] | 1.60  [1.38, 1.86] | 1.32  [1.09, 1.59] | - | 2.15  [1.87, 2.47] | 1.39  [1.22, 1.59] | 1.15  [0.99, 1.32] | - | |
| SHS | 3.21  [2.80, 3.68] | 2.20  [1.89, 2.57] | 1.94  [1.58, 2.36] | 1.32  [1.15, 1.51] | 3.24  [2.71, 3.86] | 2.16  [1.90, 2.47] | 1.78  [1.47, 2.15] | 1.35  [1.18, 1.55] | 2.85  [2.56, 3.17] | 1.85  [1.68, 2.03] | 1.52  [1.37, 1.68] | 1.33  [1.18, 1.49] | |
|  |  |  |  |  |  |  |  |  |  |  |  |  | |

NS (No symptoms), SLS (Stable-low symptoms), AOD (Adult-onset decreasing), MOD (Midlife-onset decreasing), SHS (Stable-high symptoms).

Reported as Relative Risk (RR), 95% confidence intervals (CIs)

Parameters are adjusted for sex, breastfed, mother smoked during pregnancy, gestation period, birthweight, parental social class at 0, parental education at 0, housing tenure at 7/5, access to house amenities at 7/5, crowding at age 0, 7/5 and 11/10, parents marital status at 0, maternal age at birth, mother worked in first five years, separated from child for more than a month <age 5, read to at 7/5, CM wet the bed at 7/5, had any medical conditions at 7/5, Body Mass Index (BMI) at 11/10, and cognitive ability at 7/5 and 11/10.

Wave 1 (May 2020), Wave 2 (September to October 2020) and Wave 3 (February-March 2021)

**Table S11.2: Influence of psychological distress trajectories on the RR of mental health outcomes during the COVID19 pandemic, comparing different psychological distress trajectories in the BCS70**

|  | Wave 1  RR, [95% CI] | | | | Wave 2  RR, [95% CI] | | | | Wave 3  RR, [95% CI] | | | |  |
| --- | --- | --- | --- | --- | --- | --- | --- | --- | --- | --- | --- | --- | --- |
|  | NS | EAD | AOD | MOI | NS | EAD | AOD | MOI | NS | EAD | AOD | MOI | |
| Psychological distress |  |  |  |  |  |  |  |  |  |  |  |  |  |
| EAD | 3.28  [2.50, 4.30] | - |  |  | 3.08  [2.40, 3.95] | - |  |  | 2.67  [2.18, 3.28] | - |  |  | |
| AOD | 3.90  [2.86, 5.33] | 1.19  [0.89, 1.59] | - |  | 3.73  [3.08, 4.52] | 1.21  [0.99, 1.49] | - |  | 3.15  [2.58, 3.84] | 1.18  [0.95, 1.47] | - |  | |
| MOI | 7.67  [6.15, 9.57] | 2.34  [1.91, 2.86] | 1.97  [1.51, 2.55] | - | 6.66  [5.52, 8.03] | 2.16  [1.76, 2.66] | 1.78  [1.48, 2.15] | - | 5.57  [4.68, 6.62] | 2.09  [1.78, 2.44] | 1.77  [1.46, 2.14] | - | |
| SHS | 10.79  [8.77, 13.27] | 3.29  [2.65, 4.08] | 2.77  [2.18, 3.51] | 1.41  [1.24, 1.60] | 9.31  [7.78, 11.15] | 3.03  [2.48, 3.70] | 2.50  [2.14, 2.91] | 1.40  [1.26, 1.55] | 7.57  [6.51, 8.80] | 2.83  [2.44, 3.29] | 2.40  [2.05, 2.82] | 1.36  [1.24, 1.49] | |
| Life satisfaction |  |  |  |  |  |  |  |  |  |  |  |  | |
| EAD | 0.85  [0.77, 0.93] | - |  |  | 0.84  [0.80, 0.89] | - |  |  | 0.85  [0.79, 0.91] | - |  |  | |
| AOD | 0.82  [0.75, 0.89] | 0.96  [0.85, 1.09] | - |  | 0.77  [0.70, 0.83] | 0.91  [0.82, 1.00] | - |  | 0.79  [0.73, 0.86] | 0.94  [0.84, 1.05] | - |  | |
| MOI | 0.67  [0.61, 0.74] | 0.79  [0.70, 0.90] | 0.82  [0.73, 0.93] | - | 0.63  [0.58, 0.70] | 0.75  [0.67, 0.84] | 0.83  [0.73, 0.94] | - | 0.69  [0.63, 0.76] | 0.82  [0.73, 0.91] | 0.87  [0.77, 0.98] | - | |
| SHS | 0.49  [0.42, 0.57] | 0.58  [0.49, 0.68] | 0.60  [0.52, 0.70] | 0.73  [0.62, 0.86] | 0.41  [0.37, 0.47] | 0.49  [0.43, 0.56] | 0.54  [0.48, 0.61] | 0.65  [0.56, 0.76] | 0.49  [0.42, 0.57] | 0.58  [0.49, 0.68] | 0.62  [0.53, 0.71] | 0.71  [0.59, 0.85] | |
| Loneliness |  |  |  |  |  |  |  |  |  |  |  |  | |
| EAD | 1.94  [1.61, 2.35] | - |  |  | 1.77  [1.49, 2.09] | - |  |  | 1.78  [1.55, 2.04] | - |  |  | |
| AOD | 2.00  [1.66, 2.40] | 1.05  [0.82, 1.34] | - |  | 1.94  [1.66, 2.28] | 1.06  [0.83, 1.35] | - |  | 1.89  [1.63, 2.20] | 1.05  [0.82, 1.34] | - |  | |
| MOI | 2.38  [1.98, 2.86] | 1.28  [1.03, 1.59] | 1.19  [0.99, 1.44] | - | 2.40  [2.07, 2.78] | 1.28  [1.03, 1.60] | 1.23  [1.04, 1.47] | - | 2.13  [1.87, 2.44] | 1.28  [1.03, 1.59] | 1.13  [0.94, 1.35] | - | |
| SHS | 3.09  [2.62, 3.65] | 1.69  [1.38, 2.08] | 1.55  [1.30, 1.84] | 1.30  [1.12, 1.51] | 3.43  [3.04, 3.88] | 1.66  [1.36, 2.03] | 1.77  [1.54, 2.03] | 1.43  [1.25, 1.65] | 3.09  [2.78, 3.43] | 1.69  [1.38, 2.08] | 1.63  [1.40, 1.91] | 1.45  [1.28, 1.64] | |
|  |  |  |  |  |  |  |  |  |  |  |  |  | |

NS (No symptoms), EAD (Early adult-onset decreasing), AOD (Adult-onset decreasing), MOI (Midlife-onset increasing), SHS (Stable-high symptoms).

Reported as Relative Risk (RR), 95% confidence intervals (CIs)

Parameters are adjusted for sex, breastfed, mother smoked during pregnancy, gestation period, birthweight, parental social class at 0, parental education at 0, housing tenure at 7/5, access to house amenities at 7/5, crowding at age 0, 7/5 and 11/10, parents marital status at 0, maternal age at birth, mother worked in first five years, separated from child for more than a month <age 5, read to at 7/5, CM wet the bed at 7/5, had any medical conditions at 7/5, Body Mass Index (BMI) at 11/10, and cognitive ability at 7/5 and 11/10.

**Table S12: Classification of longitudinal classes comparing CBQ with Malaise Inventory at age 16**

|  |  | Longitudinal classes with CBQ at age 16 | | | | | |
| --- | --- | --- | --- | --- | --- | --- | --- |
|  |  | NS | EAD | AOD | MOI | SHS | Total |
|  | NS | 5668 | 36 | 19 | 22 | 0 | 5745 |
| Malaise at age 16 |  | 97.8% | 3.2% | 1.8% | 2.3% | 0.0% | 56.3% |
|  | EA**D** | 66 | 1055 | 0 | 3 | 16 | 1140 |
|  |  | 1.1% | 94.7% | 0.0% | 0.3% | 1.3% | 11.2% |
|  | AOD | 20 | 0 | 1039 | 8 | 16 | 1083 |
|  |  | 0.3% | 0.0% | 97.6% | 0.8% | 1.3% | 10.6% |
|  | MOI | 42 | 0 | 2 | 919 | 14 | 977 |
|  |  | 0.7% | 0.0% | 0.2% | 95.6% | 1.1% | 9.6% |
|  | SHS | 0 | 23 | 5 | 9 | 1229 | 1266 |
|  |  | 0.0% | 2.1% | 0.5% | 0.9% | 96.4% | 12.4% |
|  |  | 5796 | 1114 | 1065 | 961 | 1275 | 10211 |
|  | Total | 100.0% | 100.0% | 100.0% | 100.0% | 100.0% | 100.0% |
|  |  | 56.8% | 10.9% | 10.4% | 9.4% | 12.4% | 100.0% |

NS (No symptoms), EAD (Early adult-onset decreasing), AOD (Adult-onset decreasing), MOI (Midlife-onset increasing), SHS (Stable-high symptoms).

**Figure S3: Five longitudinal classes of psychological distress from age 16 to 46 in the BCS70 using the Malaise Inventory at age 16**

**Supplementary references**

1. Von Hippel PT. Regression with missing Ys: An improved strategy for analyzing multiply imputed data. *Sociological Methodology* 2007; **37**: 83-117.
2. Moulton, V., McElroy, E., Richards, M., Fitzsimons, E., Northstone, K., Conti, G., Ploubidis, G.B., Sullivan, A., & O’Neill, D. (2020). A guide to the cognitive measures in five British birth cohort studies. London, UK: CLOSER.
3. Enders CE. Applied missing data analysis. New York: Guilford; 2010.
4. Musliner, K.L., Munk-Olsen, T., Eaton, W. W., Zandi, P. P. (2016). Heterogeneity in long-term trajectories of depressive symptoms: Patterns, predictors and outcomes. Journal of affective disorders, 192, 199-211. doi.org/10.1016/j.jad.2015.12.030
5. Ram, N., & Grimm, K. J. (2009). Growth mixture modeling: A method for identifying difference in longitudinal change among unobserved groups. International Journal of Behavioral Development, 33, 565–576. doi:10.1177/0165025409343765
6. Lubke, G. H., & Neale, M. C. (2006). Distinguishing between latent classes and continuous factors: Resolution by maximum likelihood. Multivariate Behavioral Research, 4, 499–532. doi:10.1207/ s15327906mbr4104_4
7. Celeux G, Soromenho G. An entropy criterion for assessing the number of clusters in a mixture model. Journal of classification. 1996;13(2):195-212.
8. Sullivan A, Brown M, Bann D. Guest Editorial: Generation X enters middle age. Longitudinal and Life Course Studies. 2015 Apr 15;6(2):120-30.
9. VanderWeele TJ, Ding P. Sensitivity Analysis in Observational Research: Introducing the E-Value. Annals of internal medicine. 2017;167(4):268-274. 29.
10. Ding P, VanderWeele TJ. Sensitivity Analysis Without Assumptions. Epidemiology (Cambridge, Mass). 2016;27(3):368-377.
